# Engineering neuroimmune regulation: biomaterial and nanotechnology platforms for neuropathology diagnosis and targeted immunomodulation

**DOI:** 10.3389/fimmu.2025.1677612

**Published:** 2026-01-09

**Authors:** Mikayla S. Jackson, Lauren G. Porter, Robert S. Oakes

**Affiliations:** 1 Department of Biomedical Engineering, University of Delaware, Newark, DE, United States; 2 Veterans Affairs Maryland Health Care System, United States Department of Veterans Affairs, Baltimore, MD, United States

**Keywords:** biomaterials, central nervous system, drug delivery & targeting, glioma, immunomodulation, immunotherapy, multiple sclerosis, neurodegeneration

## Abstract

The immune system is a potent and interwoven regulator of neuropathology in the central nervous system (CNS). For example, gliomas, neurodegenerative diseases, and autoimmune neuroinflammation all have diverse etiology and pathogenesis. Yet, there are numerous similarities in immunological dysfunction between these neuropathological conditions. Synthesizing this knowledge could inform advanced diagnostics and therapeutic platforms. For instance, detection and precision management of neuroinflammation or blood-brain barrier integrity would be broadly applicable. Understanding the targetable or controllable immune pathways of these disorders and engineering local and systemic interventions are essential for improving disease prognosis. This review integrates and contrasts the biology, detection, and treatment of the aforementioned neuropathologies. First, immunological underpinnings and recent discoveries on the role of innate and adaptive immune cells are discussed. Second, diagnostics that span molecular and nanotechnology-based platforms are detailed for enhanced imaging and screening of neuroinflammation. Last, immunomodulatory therapeutics and the precision of biomaterial platforms for neuroimmune regulation are examined. Within this section on therapeutics, both clinical and experimental approaches are stratified by CNS-localized or systemic mechanism-of-action. Overall, the integration of immunotherapies, biomaterials, advanced drug delivery platforms, and precision medicine will be critical in overcoming current treatment limitations for neuropathology that have few or no therapeutic options.

## 1 Introduction

Neuroinflammation is an immune response within the central nervous system (CNS) that arises when normal homeostasis is disrupted (1–3). The response is facilitated by local glial cells that recruit peripheral immune cells, including macrophages and lymphocytes (1). A wide range of pathological stimuli, including infections, trauma, toxic metabolites, autoimmune processes, and neurodegenerative conditions, can trigger this process. The ensuing reaction is marked by the release of pro-inflammatory cytokines and chemokines, as well as increased oxidative stress. When this reactive cascade becomes dysregulated, it can result in the disruption of the blood-brain barrier (BBB), ultimately leading to tissue damage and neuronal cell death (2, 4).

Tissue-resident glial cells are central regulators of neuroimmune activity. Microglia, the brain’s resident macrophages, act as the primary innate immune sentinels. They constantly monitor the neural environment and respond rapidly to signs of damage or dysfunction. In their resting state, microglia support neuronal health; once activated, they proliferate, change morphology, and release inflammatory mediators (3, 5). Astrocytes – the most abundant glial cell type – also mount strong responses to CNS injury. Under healthy conditions, they maintain homeostasis, support the BBB, modulate synaptic transmission, and provide metabolic substrates to neurons. During inflammation, however, they become reactive, upregulate glial fibrillary acidic protein (GFAP), secrete cytokines, and shift into distinct functional states (6–8). Recent studies describe neurotoxic astrocytes, induced by IL-1α, TNFα, and complement component C1q released from activated microglia. These cells lose key homeostatic roles and can kill neurons and oligodendrocytes. In contrast, homeostatic astrocytes secrete factors that promote neuronal survival (8, 9). Together, microglia and astrocytes orchestrate CNS inflammation, enabling tissue repair in some contexts but driving neurotoxicity when their activity becomes excessive.

This review first highlights the immunopathological basis of CNS disease by examining how innate (*e.g.* microglia, astrocytes, macrophages) and adaptive (*e.g*. T cells, B cells) immune dysfunction contribute to gliomas, neurodegenerative disorders, and autoimmunity. Next, we survey biomaterial-based diagnostic technologies like magnetic resonance imaging (MRI) contrast-enhancing probes for CNS inflammation. Finally, we review engineered immunomodulatory platforms, from local delivery to systemic carriers, that reprogram the neuroimmune environment. Each section will highlight how cellular and tissue-level insights into neuroinflammation inform biomaterial design. Emerging technologies and future directions are presented at the end, synthesizing how converging bioengineering and immunology can enable new therapies for neuropathology.

## 2 Immunological features of neuropathologies

**Figure 1:**
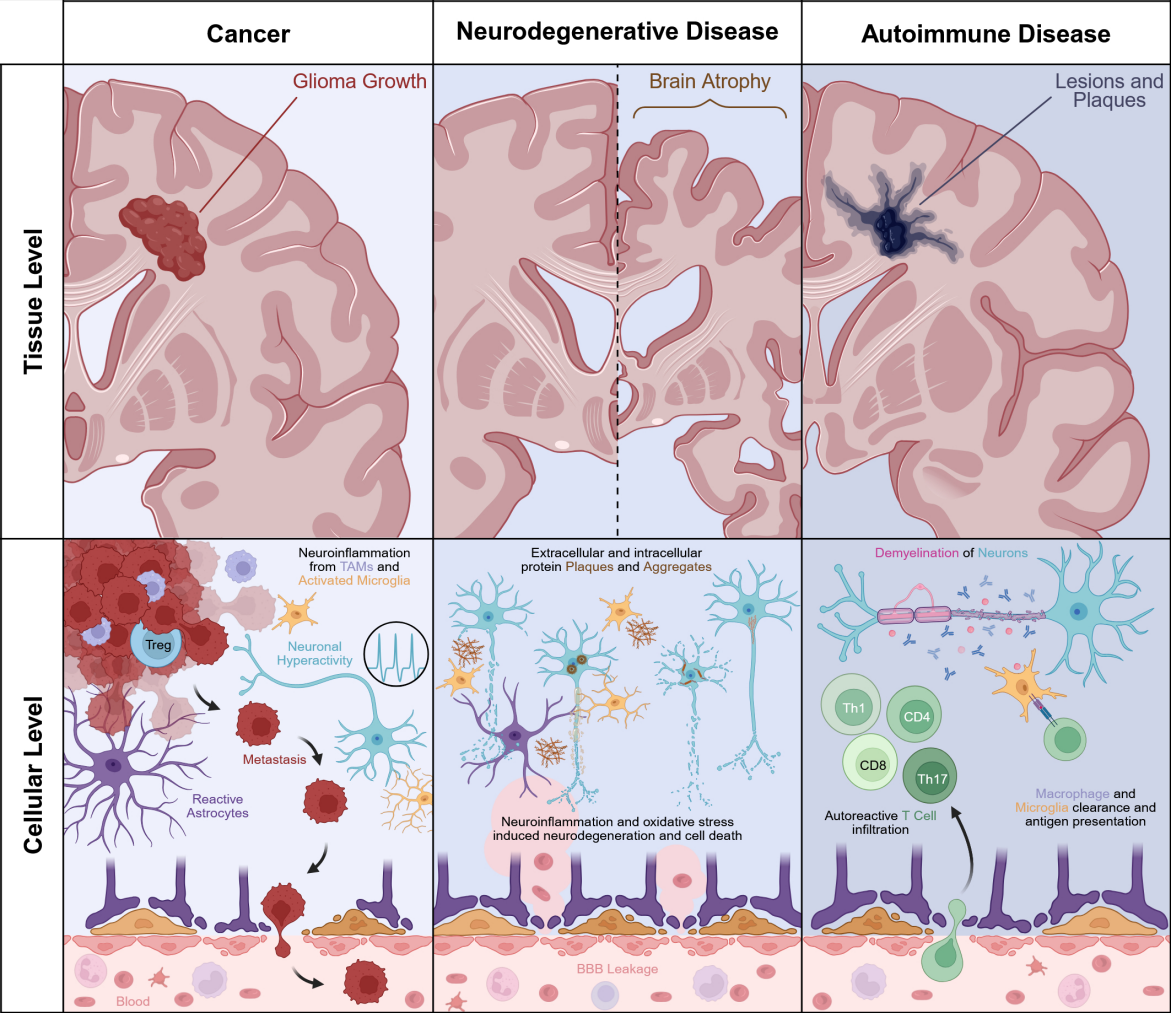
Tissue scale progression and cellular neuroinflammation of neuropathologies. Comparison of progression and immune responses of glioma growth, loss of brain mass due to neurodegeneration, and the lesions and plaques that result from the autoimmune attacks in multiple sclerosis. Neuroinflammation drives the growth and progression of gliomas, with tumor-associated macrophages, activated microglia, and regulatory T cells supporting tumor growth. Reactive astrocytes form a glial scar and facilitate metastatic tumor cells entering the BBB, as shown by the tumor cells metastasizing to the bloodstream. In neurodegenerative disease, neuroinflammation leads to neuronal loss and brain atrophy. At the cellular level, amyloid plaques and intracellular filament aggregates deposit around and within neurons, triggering microglial and astrocyte activation. Chronic neuroinflammation and oxidative stress drive progressive neuronal degeneration and apoptosis. The breach in the BBB allows for the infiltration of peripheral immune factors and neurotoxic factors in serum that exacerbate cell death. In autoimmune disease, demyelinating lesions and plaques occur in white matter tracts. T cell subsets (Th1, Th17, CD4+, and CD8+) infiltrate through a compromised BBB and attack myelinated axons along with resident microglia and infiltrating macrophages.

CNS disorders are driven by a dysregulated neuroimmune axis that disrupts neuronal homeostasis and the BBB. Persistent neuroinflammation fosters demyelination, synaptic dysfunction, and progressive neurodegeneration, highlighting the need for localized immunomodulatory interventions. The following sections describe the immunopathology and roles of immune cells in specific CNS diseases, as depicted in **Figure 1** and **Table 1**.

**Table 1:** Roles of innate and adaptive immune cells in neuropathology.

Category	Subcategory (cell/molecule)	Disease	Role in disease pathology	Ref.
Innate Immunity	Macrophages	Glioma	Skewed to an immunosuppressive phenotype; secrete IL-10, TGF-β and arginase-1; promote angiogenesis and invasion; express PD-L1 to dampen T-cell responses	(10–13)
		Alzheimer’s disease	Perivascular macrophages initially aid Aβ clearance but become chronically activated, releasing IL-1β and TNF-α that perpetuate neuroinflammation and synaptic loss	(14, 15)
		Parkinson’s disease	Infiltrate the substantia nigra; secrete TNF-α and ROS; contribute to dopaminergic neuron degeneration and α-synuclein aggregation	(16–18)
		ALS	Accumulate in the spinal cord and motor cortex; release pro-inflammatory cytokines (*e.g.* IL-1β, TNF-α) and ROS, exacerbating motor neuron loss	(14, 19, 20)
		Multiple sclerosis	Resident microglia and infiltrating macrophages phagocytose myelin, release IL-1β, TNF-α and ROS; present myelin antigen to T cells, sustaining chronic adaptive attack	(14, 21, 22)
	Microglia	Glioma	Co-opted by tumor signals into immunosuppressive, pro-tumor roles; diminished cytotoxic capacity; release growth factors and proteases that remodel the tumor stroma	(11, 13)
		Alzheimer’s disease	Cluster around Aβ plaques; chronic activation drives release of IL-1β, TNF-α and complement factors, leading to synaptic pruning and tau hyperphosphorylation	(5, 23)
		Parkinson’s disease	Activated in the substantia nigra; secrete IL-1β, TNF-α, ROS and NO; exacerbate α-synuclein aggregation and dopaminergic neuron death	(5, 24, 25)
		ALS	Early responders to motor neuron stress; release pro-inflammatory cytokines and ROS, promote neurotoxic astrocyte transition and accelerate motor neuron degeneration	(5, 8, 26)
		Multiple sclerosis	Sustain chronic lesion activity by presenting myelin antigen, releasing pro-inflammatory cytokines, and recruiting peripheral immune cells	(21, 27)
	NK cells	Glioma	Sparse infiltration; upregulate inhibitory receptors in the tumor microenvironment, limiting direct cytotoxicity against tumor cells	(28, 29)
		Alzheimer’s disease	Infiltrate the CNS in later stages; produce IFN-γ that activates microglia and exacerbates neuronal loss	(30, 31)
		Parkinson’s disease	Exhibit reduced cytotoxicity and IFN-γ production; impaired clearance of α-synuclein aggregates; contribute to persistent neuroinflammation	(32–34)
		ALS	Show increased activation but paradoxically contribute to motor neuron death via direct cytotoxicity and inflammatory cytokine release	(35–37)
		Multiple sclerosis	Reduced numbers and impaired function within lesions; fail to clear myelin debris or regulate autoreactive T cell responses	(38, 39)
Adaptive Immunity	T cells (CD4^+^, CD8^+^, Tregs)	Glioma	Infiltrate tumor margins but become exhausted (*i.e.*, PD-1, CTLA-4); skew toward Tregs that secrete IL-10 and TGF-β, fostering local immune tolerance and limiting cytotoxic killing	(40, 41)
		Alzheimer’s disease	CD4+ and CD8+ T cells cluster around Aβ plaques; secrete IFN-γ and TNF-α to activate neurotoxic microglia; show oligoclonal expansion in CSF, indicative of antigen-driven CNS response	(42, 43)
		Parkinson’s disease	Autoreactive Th1/Th17 cells respond to α-synuclein peptides; secrete IFN-γ, IL-17, and GM-CSF to disrupt BBB and recruit monocytes, driving neuroinflammation	(42–45)
		ALS	Tregs correlate with slower progression, while effector T cell infiltration and Th1 skew accelerate motor neuron degeneration	(42, 43, 46)
		Multiple sclerosis	Autoreactive Th1/Th17 cells breach the BBB and secrete IFN-γ, IL-17, and GM-CSF to drive demyelination; Tregs are insufficient or dysfunctional, permitting unchecked adaptive attack	(47–49)
	B cells	Glioma	Rare and poorly organized; produce minimal anti-tumor antibodies; functional significance remains unclear	(50, 51)
		Alzheimer’s disease	Produce autoantibodies against Aβ and tau; contribute to immune complex deposition and complement activation, which can exacerbate synaptic loss	(52, 53)
		Parkinson’s disease	Generate antibodies against α-synuclein; deposit in Lewy body regions and may activate complement, contributing to neurodegeneration	(52, 54)
		ALS	Autoantibodies against motor neuron antigens have been detected; B cell infiltration is modest but may aid antigen presentation to T cells	(55, 56)
		Multiple sclerosis	Clonal expansion in the CNS; produce oligoclonal IgG; present antigen to T cells; secrete pro-inflammatory cytokines and contribute to complement-mediated myelin damage	(57, 58)

**Figure 2:**
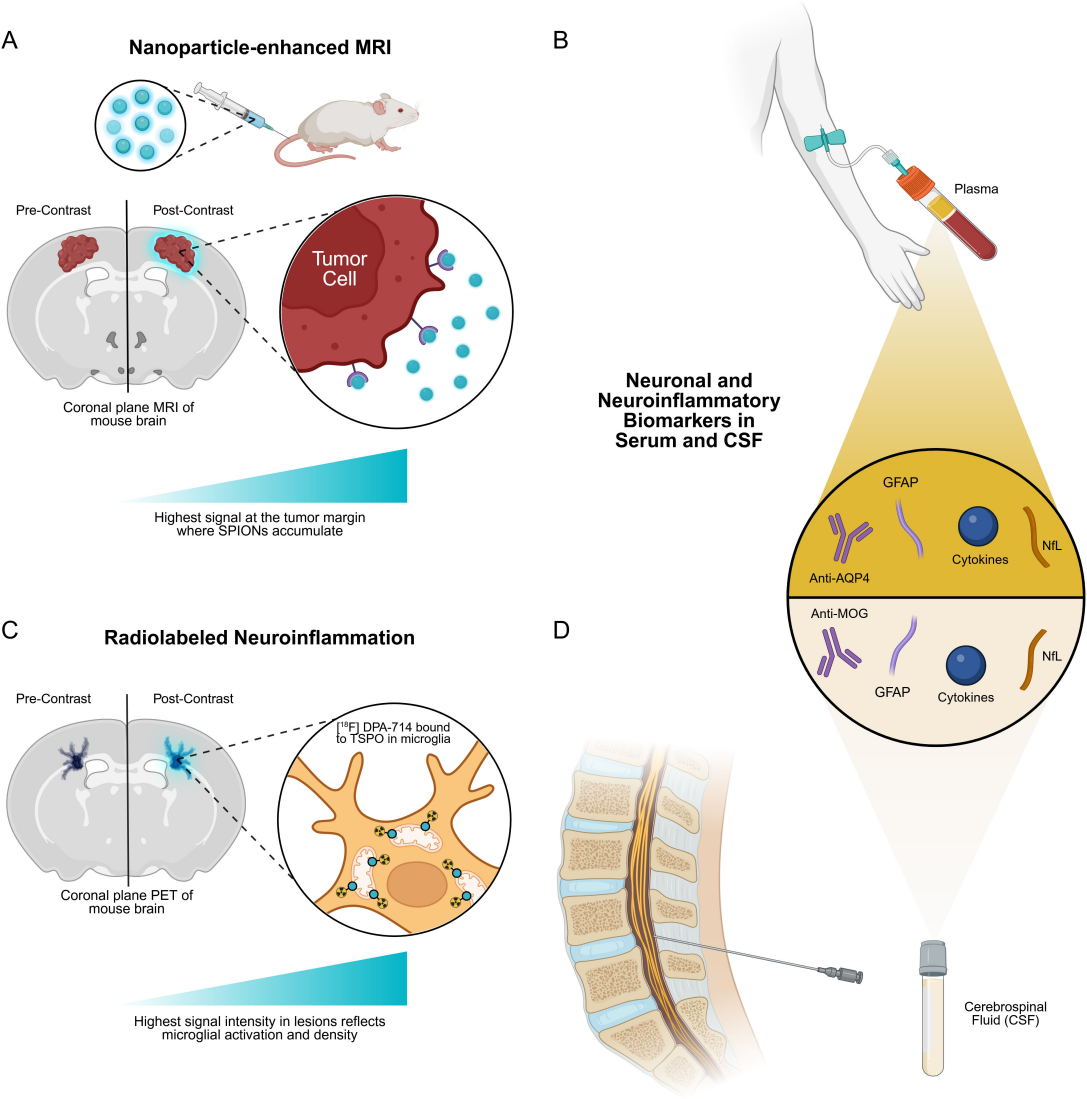
Diagnostic platforms for earlier detection of neuropathology. **(A)** Superparamagnetic iron oxide nanoparticles (SPIONs) circulate to the brain following intravenous injection. Coronal weighted MR images acquired before and after contrast enhancement show a rim around the tumor where SPIONs accumulate. At the cellular level, the mechanism involves tumor cells overexpressing target receptors that bind to surface-conjugated moieties on SPIONs, concentrating iron oxide at the tumor margin and producing a local magnetic susceptibility gradient. The color gradient indicates that the highest signal (blue) on post-contrast images corresponds to the highest SPION accumulation. **(B)** Blood and serum tests provide minimally invasive, repeatable measures that are ideal for monitoring CNS-related disorders. Common markers include neurofilament light chain (NfL) and GFAP for axonal and astrocytic injury, respectively, as well as cytokines or peripheral immune signatures that reflect ongoing inflammation. These assays are practical for tracking treatment response over time, but their interpretation can be affected by BBB integrity and peripheral inflammatory events, so results are most powerful when paired with imaging. **(C)** TSPO-targeted PET tracers are injected systemically and cross the BBB in regions of microglial activation. Coronal PET images before and after tracer administration reveal increased radiotracer uptakes in areas of neuroinflammation. This is indicative of activated microglia and the upregulation of TSPO on the outer mitochondrial membranes. **(D)** CSF obtained by lumbar puncture enables detection of CNS-specific biomarkers (NfL, GFAP, cytokines, autoantibodies) that have not crossed the BBB or reached detectable serum levels, improving sensitivity for glial activation, neuroinflammation, or early pathological processes. The combined readout of imaging and fluid biomarkers offers a powerful multimodal signature to improve early detection, differentiate disease activity from treatment or aging-related changes, and refine timing and type of interventions.

**Figure 3:**
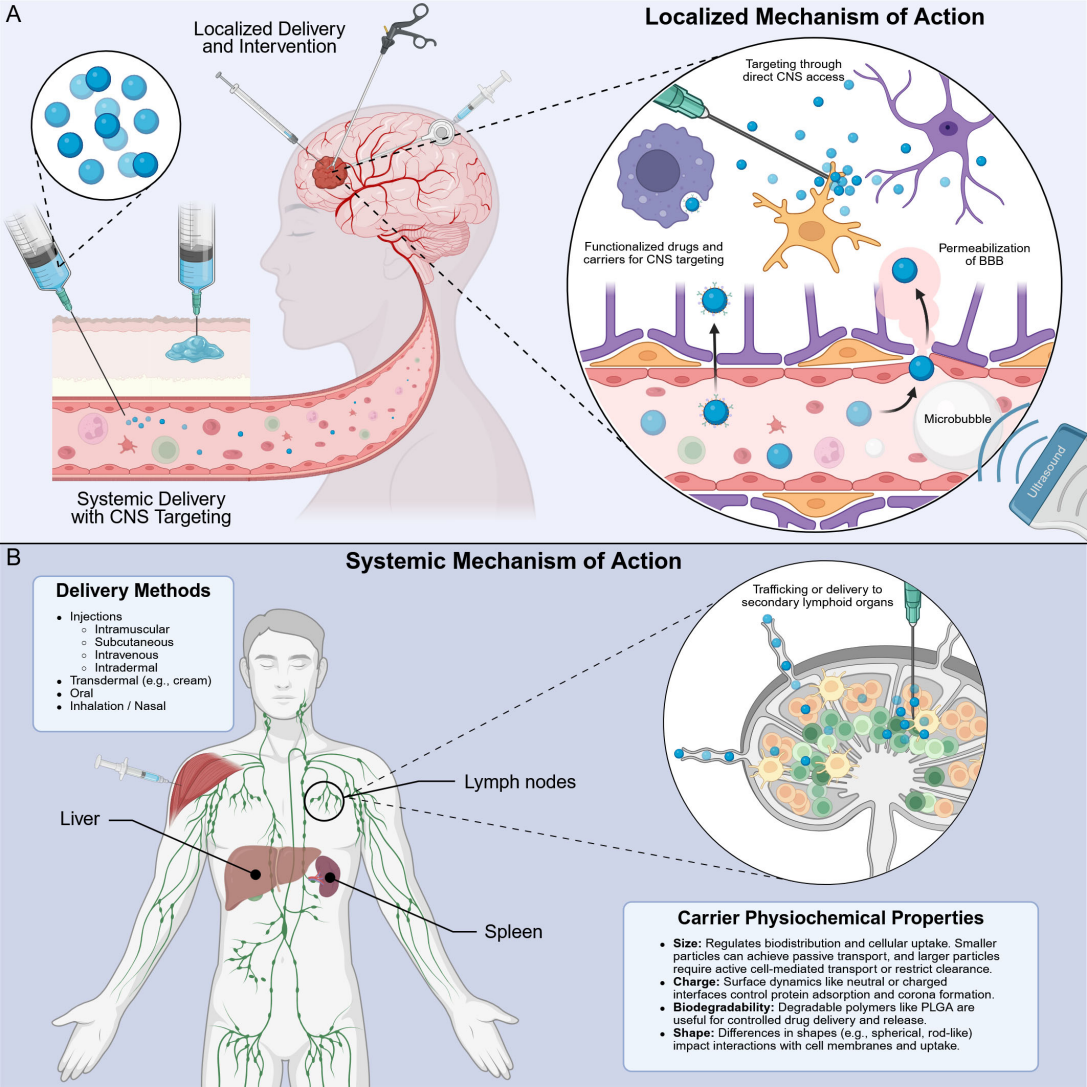
Comparison of localized and systemic mechanisms of action. **(A)** Direct implantation and injection provide various types of drug administration that immediately interact with the CNS. Additionally, functionalized therapeutics that target the CNS or strategies like focused ultrasound enlargement of microbubbles that permeabilize the BBB are routes to localize a mechanism-of-action in the CNS. **(B)** Several therapeutic strategies rely on immunological mechanisms of action that occur outside of the CNS. This is especially true for immunomodulation, where therapeutics target secondary lymphoid organs like lymph nodes following intramuscular (shown), intravenous, subcutaneous, oral, inhaled, and others delivery routes. It is also feasible to directly deliver therapeutics to lymph nodes. Biomaterial approaches, like the nanoparticles depicted above, travel via blood and lymphatics to the lymph node (shown) and other lymphoid organs for clearance or immune engagement. In the lymph node, nanoparticles and their cargo are processed to regulate systemic immune responses.

**Figure 4:**
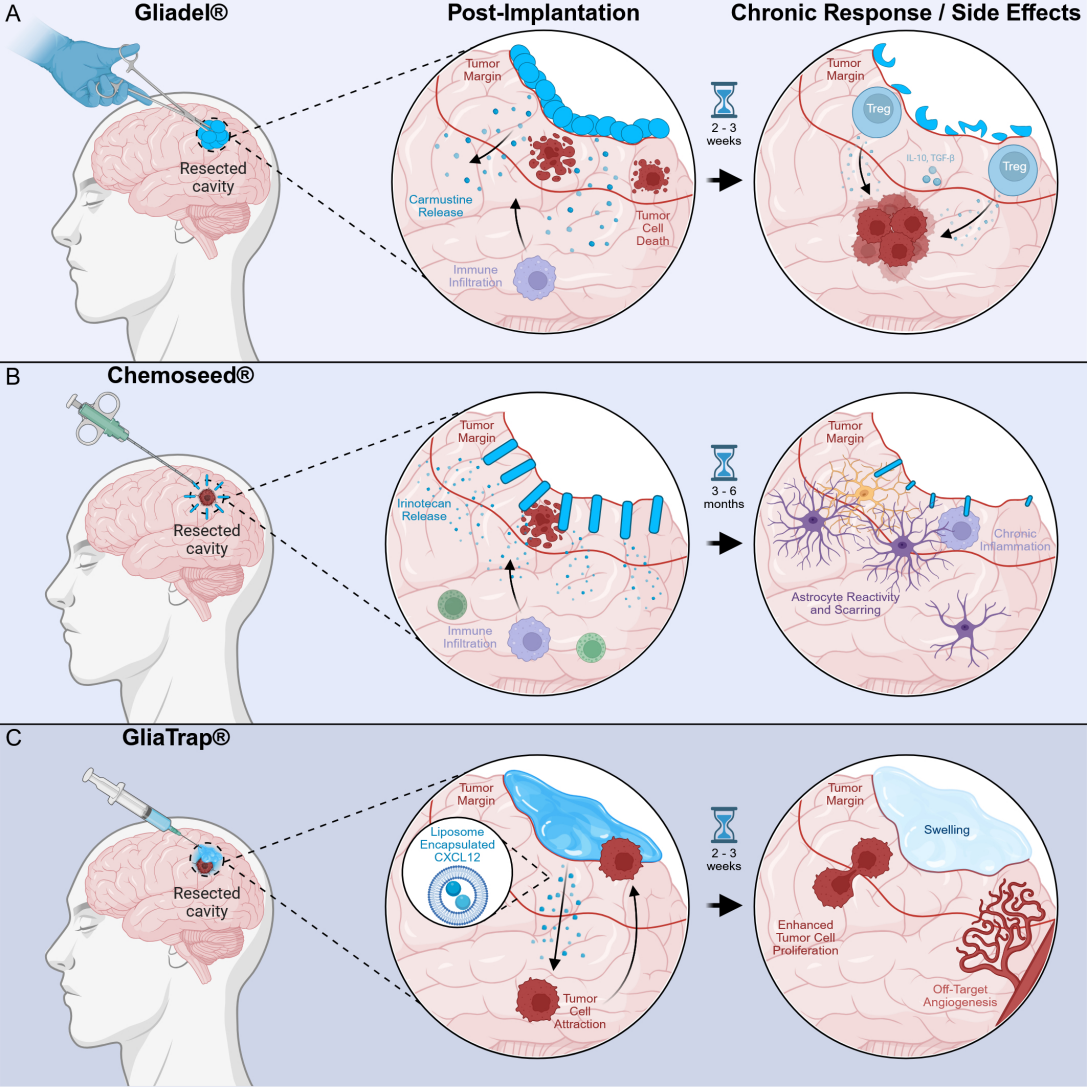
CNS-localized therapeutics for glioma. **(A)** Gliadel wafers are implanted directly into the resected tumor cavity, and they release carmustine (BCNU) locally at the tumor margin. As a result, this leads to tumor cell death and immune infiltration into the treatment area. However, after 3 weeks fragmented wafers are found in the resection cavity. This can result in chronic inflammation and the loss of chemotherapeutic release can allow upregulation of regulatory T cells (Tregs) and cytokines. Tregs induce immunosuppression to support the recurrence of gliomas. **(B)** Chemoseeds are small rods (2 mm by 6 mm) placed in the resection cavity margin to release irinotecan, a chemotherapy that targets residual glioma cells. Tumor cell death and immune infiltration are observed near the tumor margin. Over a period of 3–6 months, prolonged exposure induces astrocyte reactivity and glial scarring, which contributes to chronic inflammation that impedes recovery. **(C)** GliaTrap delivers liposome-encapsulated CXCL12, a chemokine that attracts glial cells. This acts as a trap by drawing migrating tumor cells into a controlled site, and tumor cell attraction and immune cell presence are noted near the liposome-rich area. Within a few weeks, unintended outcomes include enhanced tumor cell proliferation, swelling, and angiogenesis. These responses typically exacerbate the disease instead of controlling it.

**Figure 5:**
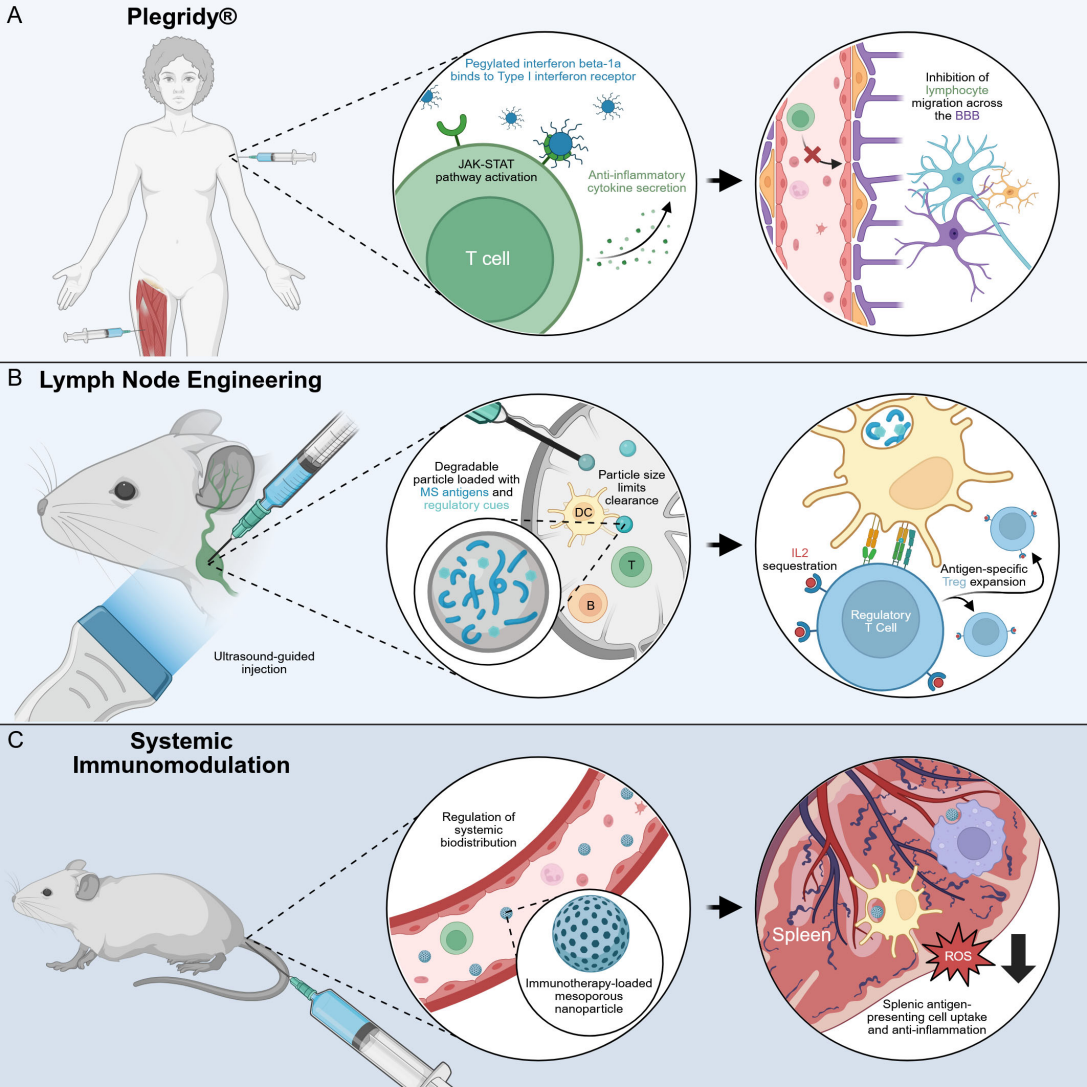
Systemic therapeutics for multiple sclerosis. **(A)** Plegridy is administered via intramuscular injection in humans, and its mechanism-of-action involves binding to the surface receptors on immune cells (T cell shown), initiating type I interferon signaling. This leads to immune modulation of cells, activation states, and cytokine expression. This reduces inflammation and immune-mediated demyelination in MS. **(B)** A lymph node engineering approach with a direct injection into the mouse lymph node to create a biomaterial-based drug depot in lymphatic tissue. Antigens are presented directly to lymph node resident APCs, which take up immunological cues. This promotes efficient antigen presentation and immune modulation, and enhances precision with minimal exposure. **(C)** Subcutaneous injection of mesoporous silica nanoparticles (MSNs) loaded with myelin antigen (MOG) and immunosuppressive cues lead to systemic immune tolerance. MSNs circulate throughout the body and reach lymphoid organs, where they promote tolerance by reducing reactive oxygen species and blunting the inflammatory response during antigen delivery.

Innate (macrophages, microglia, NK cells) and adaptive (T cells, B cells) immune cells, stratified by neuropathology with descriptions of their known role in driving initiation and/or progression. Color shading is intended to align with the general cell shading in **Figures 1**-**5**.

### 2.1 The role of neuroinflammation in glioma tumorigenesis and progression

Glioma pathogenesis involves chronic neuroinflammation, where immune dysfunction promotes tumor progression and immune evasion. This neuroinflammatory state is not simply a byproduct of glioma growth but a core driver of tumorigenesis, shaping a microenvironment that is both highly inflamed and profoundly immunosuppressive.

#### 2.1.1 Initiation and progression of gliomas

Gliomas are highly invasive primary brain tumors that create a specialized neuroinflammatory microenvironment (59). As described by Pimenta et al., up to 30-50% of glioblastoma mass can be infiltrating immune cells, mainly microglia and tumor-associated macrophages (TAMs) as well as neutrophils, myeloid-derived suppressor cells (MDSCs) and lymphocytes (60, 61). The establishment of this inflammatory tumor microenvironment (TME) is unique due to CNS immune privilege, which historically describes the brain as being shielded from systemic immune responses by the BBB (62).

Pioneering experiments in the 1920s demonstrated that the brain immune microenvironment was hospitable for tumor growth (63, 64). This neuroimmune-tumor connection is also paradoxical as links also exists between chronic inflammation and tumorigenesis, with glioma cells producing pro-inflammatory cytokines, chemokines, and growth factors that create an inflammation-enriched TME (65, 66). This inflammatory environment, paired with immunosuppression, allows the tumor to evade immune surveillance (66, 67). As a result, inflammation within the CNS fosters glioma cell proliferation and invasion, thus driving progression to high-grade malignancy. From the earliest stages of tumor formation, gliomas manipulate neuroinflammation to promote their development.

#### 2.1.2 Dysfunction of innate immune cells in gliomas

The glioma TME is dominated by innate immune cells, principally macrophages and microglia (68, 69). Macrophages are capable of phagocytosing and killing tumor cells, although glioma-derived signals skew them toward an anti-inflammatory, immunosuppressive phenotype instead of a tumoricidal state (70, 71). As a result, immunosuppressive TAMs accumulate in gliomas, where they secrete IL-10, TGF-β, arginase-1, and other factors that dampen T cell responses and support tumor growth (71, 72). TAMs also release angiogenic and tissue-remodeling molecules that facilitate glioma invasion and neoangiogenesis (68). Notably, TAMs can express immune-checkpoint ligands such as PD-L1 and enzymes that deplete nutrients needed by T cells, directly contributing to local immunosuppression (72). While TAMs retain some capacity for antigen presentation, in the context of glioma, they often present tumor antigens in a tolerogenic manner that fails to activate effective cytotoxic immunity. Overall, glioma-associated macrophages and microglia are co-opted into a pro-tumor role, suppressing anti-tumor immunity and enabling tumor expansion.

Extensive evidence from both bulk and single-cell studies, as surveyed by recent systematic reviews, confirms that TAMs and microglia comprise the dominant immune cell population in the glioma microenvironment (73). Within the glioma microenvironment, TAMs adopt phenotypes distinct from those seen in classical inflammatory responses. Such extensive reprogramming contributes to the significant intra- and inter-tumor heterogeneity observed across gliomas, as the composition and activation state of the myeloid compartment vary widely between patients and even within a single tumor. In addition to macrophages, gliomas recruit tumor-associated neutrophils (TANs) via chemokines such as IL-8 and CXCL1 (74, 75). Once in the TME, TANs secrete proteases, pro-angiogenic factors, and immunosuppressive mediators mirroring TAM activity. High-grade gliomas like glioblastoma multiforme (GBM) exhibit especially robust neutrophil infiltration, and these TANs have been implicated in tumor progression and recurrence (72, 75, 76). Besides neutrophils, other innate-like suppressor cells such as monocytic MDSCs infiltrate GBM and further inhibit T-cell function. Both monocytic and granulocytic subsets of MDSCs accumulate within the glioma microenvironment and inhibit T-cell proliferation via arginase-1 and reactive oxygen species (ROS), thereby facilitating tumorigenesis (59, 76). Bayik et al. found that monocytic MDSCs express high levels of the proliferation marker Ki-67, which renders them refractory to antibody-mediated depletion and enables continuous local immunosuppression. However, their studies also concluded that granulocytic MDSCs are enriched in the peripheral circulation and their selective depletion via antibodies confers a marked survival advantage in GBM models, underscoring their systemic role in promoting glioma progression (77).

Dendritic cells within the tumor are similarly dysfunctional or absent, which further impairs priming of anti-tumor T cells (78). Natural killer (NK) cells are present in low numbers in most gliomas and often express inhibitory receptors in the TME, limiting their cytotoxicity (28). Furthermore, the innate immune compartment in gliomas is profoundly dysregulated and, rather than mounting effective anti-tumor responses, innate cells (*e.g.*, TAMs, TANs, MDSCs) are prompted by the tumor to create an immunosuppressive, tumor-promoting niche. This innate dysfunction is a central feature of glioma immunopathology and a barrier to effective immune clearance of the tumor (78). For this reason, innate dysfunction is also a key opportunity for diagnostic and therapeutic innovations.

#### 2.1.3 Adaptive T and B cell dysregulation in gliomas

In homeostasis, malignant cells can be recognized and eliminated by adaptive immune cells. In gliomas, however, the adaptive immune response is blunted and misdirected. In particular, GBM is characterized by poor immunogenicity and a highly immunosuppressive microenvironment (59, 79). Although T lymphocytes do infiltrate gliomas, especially along invasive margins and perivascular spaces, they often exhibit an exhausted or suppressed phenotype. In murine GBM models, initial infiltration of CD8+ and CD4+ T cells is observed during early tumor development, but as tumor cells proliferate they become immune evasive, with tumor-infiltrating T cell numbers declining markedly (80). In patients, tumor-infiltrating T cells commonly express high levels of immune checkpoints (*e.g.*, PD-1, CTLA-4) and reduced cytotoxic mediators, indicating functional exhaustion. The immunosuppressive cytokines and checkpoints in the GBM TME derive not only from tumor cells, but also from TAMs and regulatory T cells (Tregs), the suppressive subset of CD4+ T cells. Furthermore, GBM recruits Tregs, which accumulate disproportionately in the tumor, produce IL-10, and sequester other cytokines to dampen cytotoxic T cell activity. Studies have found that the ratio of Tregs to effector T cells is significantly elevated in GBM versus normal brain or non-malignant inflammatory lesions, contributing to local immune tolerance of the tumor (40).

B cells and antibodies are not prominent players in most gliomas. Unlike some peripheral solid tumors, gliomas rarely induce strong humoral immune responses (81). A minority of gliomas may contain B cell aggregates, but their functional significance remains unclear (82). It is hypothesized that the profound immunosuppression in the glioma milieu not only hinders T cell function but also interferes with B cell activation (83, 84). Therefore, the adaptive immune dysregulation in gliomas is characterized mainly by T cell dysfunction. The net effect is that gliomas manage to escape adaptive immunity despite the presence of T and B cells in the CNS (40, 59).

### 2.2 Neuroinflammation in neurodegenerative diseases

Neurodegenerative diseases such as Alzheimer’s disease (AD), Parkinson’s disease (PD), and amyotrophic lateral sclerosis (ALS) are characterized by progressive loss of neurons with accompanying chronic inflammation in the CNS. In these disorders, misfolded proteins and degenerating neurons serve as triggers for innate immune activation, and there is increasing evidence for adaptive immune involvement as well.

#### 2.2.1 Initiation and progression of neurodegenerative diseases

Each neurodegenerative disease has distinct initiating pathological proteins and vulnerable neuronal populations, but a common theme is that the accumulation of protein aggregates and resulting neuronal stress elicits a sustained immune response in the brain. In AD, extracellular β-amyloid (Aβ) plaques and intracellular tau neurofibrillary tangles are the core lesions (85). Systematic reviews have revealed a significant role for neuroinflammation in AD pathogenesis and progression. Even early in the disease, microglia and astrocytes are found clustered around Aβ plaques, and elevated inflammatory mediators are detectable (86–88). A recent meta-analysis of cerebrospinal fluid (CSF) and plasma biomarkers have confirmed increased levels of IL-6, TNF-α, and other pro-inflammatory cytokines across AD cohorts, further supporting a role for persistent immune activation (89).

In PD, the initiating pathology is the misfolding of α-synuclein in dopaminergic neurons of the substantia nigra, leading to Lewy body formation and neuronal death (90, 91). This neuronal injury in PD is accompanied by notable immune changes, as the BBB becomes abnormally permeable and pro-inflammatory molecules and cells from the periphery infiltrate the midbrain (92–94). Activated microglia are observed in the affected regions even in early PD, suggesting that inflammation is intertwined with the neurodegenerative process from the start. Indeed, PD patients often have elevated levels of inflammatory cytokines systemically and in CSF, pointing to a systemic immune activation triggered by or responding to neurodegeneration (95).

In ALS, a disease of motor neuron degeneration, neuroinflammation is likewise an early feature. In both ALS patients and animal models, astrocytes and microglia become reactive even before overt motor symptoms develop (96). Moreover, BBB and blood-spinal cord barrier disruption occurs in ALS, permitting infiltration of immune cells into the spinal cord (97). Studies in presymptomatic ALS mice have shown that neuronal stress leads to microglial activation and T cell entry, indicating the immune system is engaged early in the disease course (98, 99).

Across AD, PD, and ALS, the initial activation of the immune system may have a protective intent. Microglia initially attempt to phagocytose Aβ plaques in AD, and certain immune responses may help compensate for neuronal loss. However, if the injurious stimulus is not resolved, the immune activation becomes chronic (100, 101). This chronic neuroinflammation establishes a self-perpetuating loop as neuronal damage triggers inflammation, and inflammation causes further neuronal damage. In all three diseases, evidence of this vicious cycle has been observed (1, 7).

#### 2.2.2 Dysfunction of innate immune cells in neurodegenerative disease

In homeostasis, microglia are responsible for surveying the environment and responding to injury or infection. In neurodegenerative diseases, microglia transition from a ramified resting state to an activated state characterized by amoeboid morphology, proliferative expansion, and secretion of inflammatory mediators (5). In AD, microglial activation is initially beneficial: activated microglia surround Aβ plaques and attempt to phagocytose amyloid deposits. In early stages, this can slow plaque growth and mitigate neurotoxicity. However, as Aβ plaques and tau fibrils accumulate, microglial activation becomes chronic and dysregulated. Stimulated by this ongoing accumulation, microglia produce a host of pro-inflammatory and neurotoxic factors, including nitric oxide (NO), ROS, IL-1β, TNF-α, and C1q, which can injure synapses and neurons (88, 101). This illustrates how chronic microglial activation directly contributes to core AD pathologies. Notably, genetic studies have implicated microglial dysfunction in AD risk: rare variants in microglial genes such as *TREM2*, which encodes an innate immune receptor on microglia, substantially increase AD risk, presumably by impairing microglial ability to respond to and clear amyloid. Positron emission tomography (PET) data from a meta-analysis by Pan et al. also confirms that microglial activation is an early feature of AD, and correlates with disease severity (102).Thus, when microglia are dysfunctional or overactivated, AD pathology accelerates (88).

In PD, microglia in the substantia nigra respond to degenerating dopaminergic neurons by producing pro-inflammatory cytokines and ROS, which can damage remaining neurons. Post-mortem PD brains and animal models show activated microglia in affected regions with high expression of inducible NO synthase (iNOS) and pro-inflammatory cytokines (24). These factors contribute to oxidative stress in neurons and may promote further α-synuclein misfolding, creating a deleterious feedback loop. Indeed, chronic systemic inflammation or injections of inflammatory stimulants can worsen nigral neuron loss in PD models, underlining the causative potential of innate immune activation (25).

Early in ALS, microglia may secrete neurotrophic factors or clear toxic protein aggregates, potentially playing a protective role in disease progression (14). But as ALS develops, microglia assume a pro-inflammatory, neurotoxic phenotype. For example, in mutant SOD1 ALS models, microglia upregulate surface markers like CD14 and Toll-like receptors (TLR2/4) that recognize debris from sick motor neurons, leading to activation of NF-κB and inflammasome pathways (103). PET imaging in ALS patients demonstrates widespread microglial activation in the motor cortex and spinal cord, correlating with the extent of motor neuron degeneration. Across AD, PD, and ALS, chronic microglial activation is a common pathological denominator – while originally meant to contain damage, this activation ultimately propagates disease progression when continuously stimulated (26, 104).

Astrocytes, another type of glial cell, also exhibit dysfunctional activation, known as astrogliosis, in neurodegeneration. Activated astrocytes can produce pro-inflammatory cytokines, complement components, and reactive nitrogen/oxygen species, amplifying the inflammatory environment (105). In AD and PD, astrocytes internalize proteopathic species but often become overwhelmed and contribute to spreading pathology by releasing inflammatory signals (106). In ALS, mutant SOD1 in astrocytes causes them to secrete a toxin that selectively kills motor neurons, and transplantation experiments show that wild-type astrocytes can slow ALS progression whereas mutant astrocytes accelerate it. Additionally, chronic inflammatory activation of astrocytes reduces their support functions for neurons, exacerbating neuronal dysfunction (107).

#### 2.2.3 Adaptive T and B cell dysregulation in neurodegenerative disease

Traditionally, neurodegenerative disorders were not considered to be driven by the adaptive immune system, in contrast to autoimmune diseases such as MS. However, emerging evidence indicates that T cells and, to a lesser extent, B cells participate in the neuroinflammatory aspects of AD, PD, and ALS. Their roles are complex and can be both harmful and beneficial as described below.

Brains of AD patients show an accumulation of T lymphocytes in addition to glia. Both CD4+ and CD8+ T cells have been found in AD post-mortem tissue, especially in and around amyloid plaques and tau tangle-bearing neurons. Recent studies suggest they actively contribute to pathology (42). In a 3D human AD model, the infiltration of CD8+ T cells led to heightened microglial activation and neurodegeneration, mediated by interferon-γ (IFN-γ) signaling (108). There is evidence for clonal expansion of T cells in the CSF of AD patients, indicating an antigen-driven response in the CNS. The consequence of T cell activity in AD appears to be predominantly detrimental, as T cells secrete IFN-γ and TNF, which further activate microglia to a neurotoxic phenotype and directly induce neuronal death or synapse loss. B cells and antibodies are less prevalent in AD data, but some studies have found autoantibodies against neuronal proteins in patients; their significance is unclear, and AD is not generally considered a B-cell-driven disease (109).

In PD, the link to adaptive immunity was solidified by the discovery that peripheral T cells from PD patients can recognize α-synuclein peptides presented by human leukocyte antigen molecules. Sulzer et al. showed that certain epitopes of α-synuclein act as antigenic targets for CD4+ T cells in individuals with PD, strongly suggesting an autoimmune component to the disease (110). This breakthrough explained earlier findings that T cells infiltrate the PD brain. These T cells often exhibit an activated, pro-inflammatory profile and have been observed in close apposition to degenerating dopaminergic neurons, implying direct interaction and possible cytotoxicity (110). The presence of adaptive immune cells in PD raises the possibility that the immune system mistakenly identifies stressed neurons (or aggregated α-synuclein within them) as targets. Adaptive immune dysregulation in PD appears to contribute to neuron loss: T cell infiltration and activation in the CNS correlates with more rapid disease progression. Supporting this, reducing T cell activity in PD animal models (*e.g.*, via immunosuppressants) can attenuate neurodegeneration, whereas exacerbating T cell responses can worsen neuron death (111).

ALS has the most nuanced interplay with adaptive immunity. Both T and B cells have been found in ALS-affected spinal cord and motor cortex, especially at disease onset. Uniquely, some aspects of the adaptive response in ALS might be protective. Research has shown that Tregs are key modulators of ALS progression. Patients with slower ALS progression tend to have higher levels of circulating Tregs, whereas rapidly progressing patients have fewer or dysfunctional Tregs (112). Conversely, pro-inflammatory T helper cells (Th1, Th17) and cytotoxic CD8+ T cells in ALS can contribute to motor neuron injury. B cells are less studied in ALS, but some patients exhibit circulating autoantibodies against motor neuron proteins or lipids (113). There is evidence that immunoglobulins from ALS patients can induce motor neuron damage in culture, hinting at a possible pathogenic role of B cells in at least a subset of ALS. Overall, in ALS the adaptive immune system appears to have a dual role: a well-regulated adaptive response dominated by Tregs and possibly Th2 cells may be neuroprotective, whereas a shift toward pro-inflammatory adaptive immune response with high concentrations of Th1/Th17 and cytotoxic T cells likely exacerbates neurodegeneration (114, 115).

### 2.3 Autoimmune-driven neuroinflammation

Multiple sclerosis (MS) is an autoimmune neuroinflammatory disease in which the immune system mistakenly attacks components of the CNS, leading to demyelination and neurodegeneration. MS immunopathology is characterized by peripheral immune activation, breach of the BBB, and infiltration of immune cells into the CNS, followed by an interplay of innate and adaptive immune responses that damage myelin and axons (47, 116). This section discusses MS pathogenesis with emphasis on neuroinflammation and BBB disruption, the roles of dysfunctional innate immune cells, adaptive T and B cell dysregulation, and the resultant pathological outcomes.

#### 2.3.1 Multiple sclerosis pathogenesis and BBB disruption

The initiating events in MS involve aberrant activation of autoreactive lymphocytes in the periphery. Once activated, pathogenic T cells circulate and gain entry to the CNS by crossing the BBB. BBB breakdown and immune cell infiltration into the CNS are early hallmarks of MS. Gadolinium-enhanced MRI in early MS shows focal BBB leakage even before substantial demyelination occurs, indicating that an inflammatory attack on the BBB endothelium is one of the first steps of lesion formation (117). Activated T cells express integrins and chemokine receptors that allow them to adhere to and penetrate the brain endothelial lining, which is often upregulated with adhesion molecules during MS flares. The mechanisms of BBB disruption in MS may include direct effects of cytokines, including IL-17 and TNF from infiltrating T cells, and intrinsic endothelial abnormalities. Once across the BBB, the immune cells infiltrate the CNS parenchyma and perivascular spaces, launching an attack on myelin regions (118, 119).

The pathological hallmark of MS is the formation of demyelinating plaques in the brain and spinal cord, which are areas of myelin loss with relative initial preservation of axons and associated inflammation and gliosis (47). These focal lesions are caused by the coordinated assault of immune cells: CD4+ T helper cells, CD8+ cytotoxic T cells, B cells/plasma cells, and myeloid cells all invade the CNS and contribute to myelin destruction (120). An initial demyelinating event occurs when a wave of autoreactive immune cells breach the BBB and create areas of intense inflammation that strip myelin off axons, known as demyelination, and injure myelin-producing cells, known as oligodendrocytes. The presence of immunoglobulins and complement on myelin sheaths in active lesions indicates that antibodies also play a role in the myelin damage (121). Therefore, MS pathogenesis represents a break in CNS immune privilege as peripheral immune cells access and react to CNS antigens. Over time, chronic inflammation can persist behind a repaired BBB, especially in progressive MS, but the initial establishment of neuroinflammation requires BBB disruption and infiltration. In summary, the pathogenesis of MS neuroinflammation begins with autoreactive immune cells in the periphery, proceeds through a compromised BBB that allows these cells to enter the CNS, and results in concentrated immune-mediated damage to CNS tissue (4, 120).

#### 2.3.2 Dysfunction of innate immune cells in MS

Innate immune cells within the CNS are key drivers and modulators of damage in MS. Microglia and infiltrating monocyte-derived macrophages are the predominant innate cells in active lesions, where they accumulate in large numbers and contain abundant myelin debris, reflecting their central role in demyelination (122). Early in lesion development, microglia are rapidly activated by danger signals from infiltrating T cells and injured myelin. Once activated, they upregulate antigen-presentation and costimulatory pathways, thereby re-stimulating myelin-reactive T cells within the CNS and sustaining the adaptive response. Microglia and macrophages also directly mediate tissue injury through the release of pro-inflammatory cytokines (e.g., IL-1β, IL-6, TNF-α) and reactive mediators that contribute to oligodendrocyte and axonal damage (6, 123). In pattern II lesions, antibody and complement deposition enhances macrophage-mediated myelin phagocytosis, highlighting their active participation in demyelination rather than passive debris clearance (123). Multiple independent histopathological and transcriptomic studies report that microglia and macrophages in MS lesions adopt activated, phagocytic phenotypes, supporting a central role for innate myeloid cells in demyelination and lesion dynamics (124).

Innate cells further amplify inflammation by producing chemokines that recruit additional leukocytes into the CNS. Astrocytes and microglia release CXCL10, CCL2, and related signals that attract T cells, monocytes, and neutrophils, promoting lesion expansion. A rim of activated microglia at the edge of acute lesions is commonly observed and is thought to drive outward propagation of myelin damage into adjacent tissue (122).

Despite these pathogenic roles, microglia and macrophages also contribute to repair. They remove myelin debris – a prerequisite for effective remyelination – and can secrete anti-inflammatory cytokines and trophic factors that support tissue recovery. Subsets of these cells adopt pro-repair phenotypes during lesion resolution; however, in progressive MS this reparative program becomes insufficient, and chronically activated innate cells predominantly sustain neurotoxic inflammation (47, 120).

Overall, dysregulated innate immune activation in MS results in microglia and macrophages that both perpetuate inflammation and directly damage CNS tissue, contributing to neurodegeneration even when overt relapses decline.

#### 2.3.3 Adaptive T and B cell dysregulation in MS

MS is fundamentally driven by an aberrant adaptive immune response against the CNS. The cornerstone of MS immunopathology is the dysregulation of T lymphocytes. MS has long been considered a CD4+ T cell-mediated disease, based on the Th1-driven experimental autoimmune encephalomyelitis (EAE) model and the association of MS with class II human leukocyte antigen genes (116). Indeed, Th1 cells specific for myelin antigens produce IFN-γ and lymphotoxin, which activate macrophages and microglia in lesions and directly cause demyelination. Additionally, Th17 cells, a subset of CD4+ T cells characterized by IL-17 production, are now known to play a critical role in MS progression (125). Th17 cells are highly pathogenic in MS and EAE, helping breach the BBB and recruit neutrophils. In MS patients, the quantity of Th17 cells are often elevated and secrete GM-CSF, a cytokine that is crucial for mobilizing monocytes and fueling the inflammation in the CNS (126). A dysregulation favoring pro-inflammatory Th1/Th17 cells over regulatory mechanisms is evident in MS. Autoreactive T cells in MS show resistance to regulation and heightened effector functions. There is also evidence for CD8+ T cell involvement as CD8+ T cells outnumber CD4+ T cells in active MS lesions. These CD8+ T cells can directly attack oligodendrocytes or neurons presenting myelin peptides on major histocompatibility complex (MHC) class I. Oligodendrocyte death in some lesions appears consistent with cytotoxic T cell attack (*e.g.*, granular deposition of granzyme B in oligodendrocytes). The presence of clonally expanded CD8+ T cells in MS lesions suggests a persistent antigen-driven cytotoxic response within the CNS (127).

Counterbalancing the effector T cells are Tregs, which prevent autoimmune reactions in healthy individuals. In MS, Tregs are present but often functionally impaired with reduced suppressive capacity or may be quantitatively insufficient relative to effector T cells (128). This breakdown in self-tolerance permits autoreactive T cells to cause damage. Restoring Treg function is a therapeutic aim to rein in the autoimmune attack.

B cells also play multifaceted roles in MS. Once overlooked, B cells are now known to be central to MS pathology, as evidenced by the remarkable efficacy of B cell depletion therapies *(e.g*., anti-CD20 monoclonal antibodies) (126). Antibodies produced by plasma cells in the CNS contribute to demyelination. Most MS patients have oligoclonal immunoglobulin G (IgG) bands in their CSF, indicating a persistent B cell response in the CNS (126). These antibodies likely bind myelin or other neural antigens and, together with complement, mediate myelin destruction. However, B cells are not just sources of antibodies but also function as antigen-presenting cells and cytokine producers. Memory B cells in particular form an active immune axis in MS, trafficking between blood, lymphoid organs, and the CNS (52, 57). Within lymph nodes, B cells can present myelin antigens to autoreactive T cells, effectively licensing these T cells to enter the CNS and cause damage (57). B cells can thus amplify T cell and innate immune pathology independent of antibody production. Conversely, B cells also can have regulatory subsets (*i.e.*, producing IL-10), but MS patients may have defects in these regulatory B cell populations.

The dysregulation of T and B cells in MS is also evident in the loss of peripheral tolerance checkpoints. Healthy individuals may have T and B cells that recognize myelin, but these are kept in check by deletion or anergy (129). In MS, due to genetic or environmental factors, these checkpoints fail. Autoreactive T and B cells expand in the periphery and memory cells persist. When these cells reencounter myelin antigens in the context of inflammation, they become pathogenic effectors (130).

## 3 Diagnostic platforms for early detection of neuropathologies

Early detection of CNS disorders is essential because pathological changes often occur long before symptoms emerge. Detecting disease at a preclinical or early symptomatic stage enables interventions when therapies may still alter disease trajectory, preserve function, and improve patient outcomes. Emerging studies have demonstrated robust predictive accuracy well before clinical diagnosis, offering pathways to early intervention, patient stratification, and enrollment in disease-modifying trials.

### 3.1 Nanotechnology for precise glioma diagnosis

Despite advances in surgical, chemotherapeutic, and radiotherapeutic treatment regimens, the prognosis for patients with glioma, especially GBM, remains dismal (131, 132). Central to the challenge of improving glioma outcomes is the fact that these tumors often evade early clinical detection due to their highly infiltrative nature (83). Recent years have witnessed a paradigm shift in understanding glioma pathogenesis, as neuroinflammation and immune microenvironment components are now recognized not merely as bystanders, but as fundamental drivers of glioma initiation, progression, and therapeutic resistance (133).

The detection of gliomas at an early, asymptomatic stage confers significant clinical benefits. Smaller tumor volume at diagnosis facilitates maximal surgical resection, which has consistently been associated with improved survival and preservation of neurological function (134). The earliest hallmarks of glioma are mediated by neuroinflammatory processes, and these phenomena can often precede radiological or clinical tumor manifestation. This positions them as promising candidates for sensitive, noninvasive early detection strategies (134, 135). Given this urgent need, technologies like superparamagnetic iron oxide nanoparticles (SPIONs) have emerged as a targeted approach for their early detection.

Conventional clinical imaging methods for glioma, specifically those relying on gadolinium-based contrast agents, are impeded by several limitations. As shown in **Figure 2A**, SPIONs at their core consist of magnetite nanocrystals typically 10–30 nm in diameter, coated with biocompatible polymers or targeted ligands (136, 137). The supermagnetism creates local magnetic field gradients, and these accelerate transverse proton relaxation, producing hypointense contrast on T2-weighted MRI sequences. Both transferrin and lactoferrin conjugated SPIONs surpass many clinical agents and enable the detection of submillimeter tumor foci (137, 138). These nanoparticles also exploit the enhanced permeability and retention of the leaky BBB that characterizes glioma vasculature, and extravasate into perivascular tumor regions that are invisible to small-molecule gadolinium chelates. Once in the interstitial space, their size and surface coatings prolong retention, sustaining contrast for up to 48 hours post-injection in rodent glioma models (137).

Conjugating SPION surfaces with transferrin or lactoferrin exploits overexpressed receptors on glioma cells and the BBB (136–138). Transferrin-SPIONs (Tf-SPIONs) bind transferrin receptors (TfR), while lactoferrin-SPIONs (Lf-SPIONs) engage lactoferrin receptors (LRP1), facilitating receptor-mediated endocytosis into tumor cells. *In vitro*, C6 glioma cells internalize conjugated SPIONs 2-fold to 3-fold more than non-targeted particles, yielding pronounced signal drops on T2-weighted scans. *In vivo*, receptor-targeted SPIONs accumulate intracellularly, as confirmed by Prussian blue staining, and demarcate infiltrative margins unseen with conventional agents (137, 138). By integrating superparamagnetic cores with tailored surface ligands, SPIONs transcend the limitations of existing MRI agents, achieving high sensitivity, specificity, and durable contrast for early glioma detection. Their dual passive and active targeting mechanisms hold promise for translating into clinical protocols that shift glioma management toward proactive, image-guided intervention.

In addition, complementing advanced MRI and molecular imaging with minimally invasive fluid biomarkers may improve discrimination between true tumor progression and treatment-related changes, while also providing earlier readout of tumor-associated neuroaxonal and glial injury (**Figure 2B**). Blood and CSF assays for neurofilament light chain (NfL) and GFAP increase with CNS tissue injury and have been evaluated across CNS tumors and other neurological diseases (139). Recent glioma biomarker studies advocate multimarker liquid-biopsy panels to increase sensitivity for active disease and to track dynamics after surgery or chemoradiation. Integrating serial fluid measures with SPION/targeted MRI or PET may therefore yield a combined imaging and biomarker signature that improves early detection, helps distinguish progression from treatment effects, and supports treatment timing decisions (139, 140).

### 3.2 Imaging of innate immunity for early neurodegenerative diagnosis

Early diagnosis and intervention in neurodegenerative diseases such as ALS, AD, and PD are increasingly recognized as critical factors for improving patient outcomes. As mentioned previously, a commonality among these disorders is the pivotal role of neuroinflammation in disease initiation and progression. Elevated or dysregulated neuroinflammation leads to deleterious effects, including the release of cytotoxic cytokines, oxidative damage, and synaptic dysfunction, all of which exacerbate disease progression (141).

Neuroinflammation has been difficult to assess with specificity and sensitivity, and advances in molecular imaging, particularly PET with radiotracers that bind the 18kDa translocator protein (TSPO), have made it possible to visualize and quantify these neuroinflammatory processes in living brains (**Figure 2C**) (142).

In the healthy brain, TSPO is expressed at low levels, primarily in glial cells (*e.g.* astrocytes and microglia), steroidogenic cells, and to a lesser extent in vascular endothelial cells. TSPO is functionally implicated in cholesterol transport, mitochondrial respiration, apoptosis regulation, and cellular stress responses (143, 144). Neuroinflammation, whether triggered by protein aggregates, neuronal injury, or neurodegenerative cascades, induces pronounced upregulation of TSPO, especially in activated microglia and reactive astrocytes. This upregulation is not only a marker of immune cell activation, but also serves as a functional participant in the immune response, modulating cell survival, cytokine production, and peripheral immune cell migration (143, 144).

Preclinical and clinical studies consistently demonstrate that [18F]DPA714 PET provides sensitive detection of neuroinflammation across ALS, AD, and PD, enabling early diagnosis and monitoring of disease progression.

Furthermore, in neurodegenerative disorders, plasma and CSF inflammatory biomarkers – notably YKL-40, TREM2, GFAP, and inflammatory cytokines – provide complementary information to TSPO-PET and structural imaging (89). YKL-40 and TREM2 track astrocytic and microglial activation respectively and have been associated with disease stage in Alzheimer’s disease (102, 145). Plasma GFAP has shown sensitivity to early astrocytic/amyloid pathology, and CSF/serum cytokine profiles correlate with imaging measures of neuroinflammation in several cohorts. These fluid markers can be sampled longitudinally with minimal invasiveness, and combining them with TSPO-PET or other microglial/astrocytic imaging readouts yields a multimodal profile that better characterizes active neuroinflammatory states and may inform anti-inflammatory or disease-modifying treatment strategies (145).

#### 3.2.1 Neuroinflammatory imaging in amyotrophic lateral sclerosis

In SOD1G93A transgenic mice, [18F]DPA714 uptake in skeletal muscle rises significantly at presymptomatic stages, reflecting upregulated TSPO in myocytes independent of leukocyte infiltration. Although brain uptake is modest early on, tracer binding in motor cortex and brainstem increases as disease advances, mirroring human ALS neuroinflammation and expanding diagnostic reach to neuromuscular involvement (142, 146, 147).

PET scans in ALS patients show elevated [18F]DPA714 uptake in the motor and frontal cortices, corticospinal tracts, brainstem, and thalamus of ALS patients versus controls, mapping a widespread but regionally specific inflammatory signature. Multicenter pooling of second-generation TSPO tracers, including [18F]DPA714, enhances sensitivity and reproducibility, facilitating ALS biomarker development for trials (146).

#### 3.2.2 Neuroinflammatory imaging in Alzheimer’s disease

Longitudinal PET studies in APP/PS1 mice – a transgenic mouse model of AD – reveal progressive increases in cortical and hippocampal [18F]DPA714 binding from 12–13 months onward, correlating with immunohistochemistry showing an increase in Iba-1+ microglia. Competitive blocking with the first-generation ligand PK11195 confirms tracer specificity for TSPO sites, validating [18F]DPA714 as a reliable proxy for *in vivo* microgliosis in preclinical AD models (146).

At prodromal and mild cognitive impairment stages, AD patients display heightened [18F]DPA714 binding in the temporo-parietal cortex, posterior cingulate, and precuneus, which are regions vulnerable to early amyloidosis. Longitudinal data link high baseline TSPO binding to slower cognitive decline and reduced amyloid accumulation, suggesting a nuanced, potentially protective microglial response in early AD (141).

#### 3.2.3 Neuroinflammatory imaging in Parkinson’s disease

Rats overexpressing mutant α-synuclein exhibit early, region-specific ([18F]DPA714) binding increases in the substantia nigra and striatum prior to overt dopaminergic neuron loss and motor deficits, indicating that PET-detected microglial activation anticipates classical PD pathology (146).

Early PD cohorts imaged with [18F]DPA714 exhibit increased binding in the substantia nigra, caudate, and thalamus, correlating with dopaminergic deficits on 18F-DOPA PET but not disease duration or severity scores, implying that neuroinflammation tracks discrete pathological processes. Stratifying by the rs6971 TSPO polymorphism reveals higher tracer binding in high-affinity binders (HAB), underscoring the need for genotype-informed analysis in clinical trials (146). Early diagnosis before symptom onset is proposed for guiding trial inclusion and precision medicine stratification. Longitudinal monitoring of neuroinflammatory responses to immunomodulatory therapies would also offer quantitative biomarkers of efficacy and surrogate endpoints for clinical trials. By enabling noninvasive, quantitative imaging of neuroimmune dynamics, [18F]DPA714 PET stands as a transformative biomaterial-based technology for early detection, stratification, and therapeutic monitoring across ALS, AD, and PD.

### 3.3 Detecting and monitoring autoimmune neuroinflammation in MS

Neuropathologically, MS lesions are surrounded by a rim of activated microglial cells, which is more prevalent in patients with progressive MS (147, 148). Microglial accumulation in white matter causes oxidative stress and neuronal damage, suggesting innate immune activation underlies tissue damage from the earliest stages. This indicates that microglial activation is an important indicator of tissue damage and candidate for early stage diagnostics (148).

Conventional MRI is insensitive to such diffuse inflammation. Particularly in progressive MS, active inflammatory lesions detected by MRI are rare, and instead, patients have widespread low‐grade inflammation behind an intact BBB (149). By contrast, PET imaging with TSPO ligands can visualize molecular inflammation *in vivo*. In CNS neuroinflammation, TSPO is highly expressed on activated microglia, and to a lesser extent on astrocytes and macrophages. PET radioligands for TSPO therefore serve as markers of microglial activation. In MS, TSPO-PET has revealed diffuse and focal innate immune activation not seen on MRI (148, 149).

The TSPO label fluorination introduced previously extends the radionuclide half-life (~110 min) and allows transport to PET centers without cyclotrons. Compared to first-generation ligands, [18F]-DPA-714 has higher brain uptake, higher signal‐to‐noise ratio, and lower nonspecific binding (149). Importantly, [18F]-DPA-714 PET has been shown to reliably identify increased focal and diffuse neuroinflammation in progressive MS when quantified by binding‐potential. In one proof‐of‐concept study, patients with primary or secondary progressive MS showed significantly elevated [18F]-DPA-714 binding in white-matter lesions and even normal-appearing tissue relative to controls (146, 147, 149).

Animal models have confirmed that [18F]-DPA-714 PET sensitively tracks demyelination-associated inflammation. In one mouse model of toxic demyelination, known as the cuprizone model, PET and MRI were used in tandem to follow immune cell dynamics (150). After 4 weeks of cuprizone, mice showed peak [18F]-DPA-714 uptake in the corpus callosum and hippocampus, coinciding with maximal demyelination. Uptake then declined by 6 weeks as remyelination occurred. Autoradiography and immunofluorescence validated that [18F]-DPA-714 distribution matched TSPO levels. Interestingly, microglia predominantly expressed TSPO at peak demyelination, whereas astrocytes were the major TSPO source during remyelination (150). This work shows that [18F]-DPA-714 PET noninvasively captures the regional and temporal profile of neuroinflammation in MS‐like pathology.

Recent clinical studies using [18F]-DPA-714 PET have offered new insights into MS lesion pathology and progression. In a cohort of 36 MS patients (relapsing‐remitting, secondary‐ and primary‐progressive), Bodini et al. used [18F]-DPA-714 PET to classify each white-matter lesion by innate immune content (148). They found that the majority of non-enhancing lesions harbored chronic inflammation, where 53% were homogeneously active (*i.e.*, inflamed center) and an additional 6% were rim-active (*i.e.*, inflamed periphery). In other words, nearly 59% of lesions contained a TSPO-positive component, whereas only 41% were inactive. Crucially, the number of PET-active lesions strongly predicted future neurodegeneration, as more active lesions at baseline correlated with greater cortical atrophy and worsening disability over 2 years. Bodini et al. concluded that an unexpectedly high proportion of MS lesions have a smoldering component, which predicts atrophy and clinical progression. This suggests that even after an acute flare, most MS plaques develop chronic inflammation that drives long-term damage (151).

Like the neuropathology above, these imaging modalities can be integrated with serum and CSF biomarkers (**Figure 2D**). For CNS autoimmune syndromes, well-validated disease-specific fluid biomarkers already guide diagnosis and management: serum and CSF anti-AQP4 and anti-MOG autoantibodies are clinically established for discriminating neuromyelitis optica spectrum disorder (NMOSD), MOG-antibody disease and MS-like presentations, and serial antibody titers sometimes correlate with relapse risk and radiological activity (152, 153). When combined with imaging readouts, antibody status, and dynamic fluid measures provide a more comprehensive view of lesion activity versus chronic inactive disease, aiding decisions on escalation or de-escalation of immunotherapy and helping to time interventions to periods of active inflammation (152–154).

PET imaging of markers prevalent in innate immune cells has emerged as a powerful neuroinflammation imaging tool in MS research. Animal and human studies confirm that it sensitively detects microglial activation during demyelination. In MS patients, it uncovers a high burden of chronic inflammation, often missed by MRI, that predicts neurodegeneration. These features make [18F]-DPA-714 PET a compelling biomaterial-based diagnostic strategy for early MS detection. By visualizing innate immune activity at the molecular level, it offers the potential to recognize this disease and guide timely treatment before disability advances.

## 4 Therapeutic interventions and platforms for modulating neuroinflammation

Both local and systemic biomaterial-based strategies have emerged to modulate neuroinflammation in CNS disorders, offering complementary advantages in delivery precision, immune cell targeting, and sustained release. **Figure 3** compares these therapeutic modalities, highlighting their administration routes, mechanisms of action, and immunomodulatory profiles.

### 4.1 CNS-localized mechanism of action

Neuroinflammation is a central driver of neuronal injury in various disorders, yet systemic therapeutics often suffer from poor BBB penetration and off-target effects. Biomaterial-based local intervention strategies, ranging from various delivery routes and mechanisms of action, offer spatially controlled and sustained delivery of immunomodulators directly within the CNS. This enables precise neuroimmune modulation at sites of disease.

#### 4.1.1 Tumor resection cavity implants and injectables

##### 4.1.1.1 Gliadel^®^ wafer implants

Gliadel wafers have become one of the most notable successes in biomaterial-based drug delivery systems and local chemotherapies, as clinical trials in newly diagnosed and recurrent glioblastoma demonstrate survival benefits when added to standard therapies such as surgery, radiotherapy, and temozolomide. Their success has established the wafer as an FDA-approved adjunct for high-grade gliomas (III-IV) (155). The wafers are loaded with the chemotherapeutic carmustine, also known as BCNU, and are biodegradable polymer discs placed into the resection cavity following surgical removal of a high-grade glioma (155, 156). This local delivery strategy was developed to bypass the BBB and achieve high intratumoral BCNU concentrations while limiting systemic toxicity. Upon implantation, the polyanhydride matrix hydrolyzes in brain fluid, gradually releasing BCNU and its polymer monomers into the surrounding parenchyma (**Figure 4A**). BCNU blocks DNA replication and induces apoptosis in dividing tumor cells. Since the drug release is localized, peak BCNU levels in adjacent brain are cytotoxic to residual glioma cells, whereas systemic exposure remains low. Over weeks, the wafer biodegrades about 70% to innocuous fragments (155, 157).

The implant also elicits local innate immune activity. Gliadel-associated tumor cell death releases damage signals and tumor antigens that recruit microglia and infiltrating macrophages to the cavity. These phagocytic cells clear debris and secrete cytokines, contributing to an inflammatory environment (156). Postoperative MRI often show transient reactive edema, indicated by FLAIR/T2 changes, around the wafer. Notably, one series reported that patients with a temporary surge in peritumoral FLAIR signal, indicating increased inflammation, had longer survival, which suggested a wafer-induced immune activation. This finding implies that the Gliadel wafer can modulate the neuroimmune environment (158). While primarily cytotoxic, it may also provoke an immune/inflammatory response in the tumor bed. Glial scar formation around the implant is generally mild, but reactive astrocytes and microglia cluster at the site. Thus, Gliadel wafers combine local chemotherapy with a degree of local immunomodulation, although this is not an intended design parameter (159, 160).

Despite benefits, Gliadel implantation can cause adverse local effects. The most clinically important is cerebral edema. Significant pericavity edema can develop, sometimes rapidly increasing intracranial pressure. Case reports highlight that symptomatic edema after wafer placement can be profound and even fatal if not managed. This edema is often refractory to corticosteroids, reflecting necrosis and inflammation rather than pure vasogenic swelling. Other neurologic side effects include seizures from local irritation and focal deficits (155, 156, 161).

Implantation can also interfere with wound healing, as Gliadel-treated patients showed higher incidences of CSF collection and wound breakdown than controls. Surgical site infection is notably more common in Gliadel-treated patients, with wound dehiscence and delayed healing occurring in approximately 10–20% of cases. In one review, about 3% of Gliadel-treated patients needed repeat surgery specifically for complications such as cyst formation, hydrocephalus, or wound necrosis. Rarely, chronic subdural hygroma or symptomatic cysts develop in the resection cavity. Thus, implants demand meticulous surgical technique and wound care (155–157).

Gliadel wafers represent an innovative local chemotherapy biomaterial in neuro-oncology. Moreover, the implementation of Gliadel wafers has indicated a potential and need for regulating immune responses for extended periods of time. Ongoing research into biomaterials and immune modulation aims to enhance the therapeutic efficacy of Gliadel wafers while minimizing these adverse effects, paving the way for more refined local intervention strategies that better modulate neuroinflammation in gliomas.

##### 4.1.1.2 ChemoSeed

ChemoSeed is an innovative implantable biomaterial designed to deliver localized chemotherapy within the resection cavity of high‐grade gliomas (**Figure 4B**). Preclinical data in glioblastomas showed that a single implant markedly reduced local recurrence and produced a median survival benefit, while pharmacokinetic studies showed therapeutic intraparenchymal temozolomide levels for over four weeks with minimal systemic exposure. Mechanistically, ChemoSeed pairs a biodegradable polycaprolactone scaffold with an array of biodegradable poly(lactic-co-glycolic acid) (PLGA) micro-reservoirs loaded with temozolomide to generate a staggered, multi-phase release that aims to match the repopulation kinetics of a residual infiltrative tumor (162, 163).

The underlying mechanism of ChemoSeed relies on coordinated features. The porous scaffold geometry, engineered via 3D printing, promotes tissue integration while allowing CSF to perfuse the micro-reservoirs. Second, the PLGA microspheres are formulated with distinct lactic‐to‐glycolic acid ratios which control their degradation rate and thus schedule temozolomide release in discrete pulses. Third, the scaffold surface is functionalized with arginine‐glycine‐aspartate peptide motifs to discourage the adhesion of nonmalignant glial cells, thereby biasing microsphere uptake toward invading tumor cells (162, 163).

In preclinical studies employing orthotopic glioblastoma xenografts in rats, a single ChemoSeed implant reduced local recurrence by 75% relative to systemic chemotherapy alone (164). Survival studies revealed a median extension of 30% with histological sections demonstrating concentrated apoptosis within the vicinity of the implant and preservation of surrounding healthy cortex. Pharmacokinetic analyses confirmed therapeutic intraparenchymal temozolomide concentrations for over four weeks coupled with negligible systemic exposure.

Safety evaluations in both rodent and porcine models underscore ChemoSeed clearance mechanisms, which occur without evidence of local inflammation or fibrosis after eight weeks (162, 163). The programmable release profile and minimal off‐target toxicity of ChemoSeed position it as a powerful adjuvant to surgical excision. Its scaffold can be easily tailored to incorporate synergistic agents such as antiangiogenic molecules or immunomodulators, thereby offering a flexible platform for combination strategies against highly invasive gliomas. Continued optimization of reservoir spacing and polymer composition is underway to refine spatial control of drug delivery and maximize therapeutic index (162–164).

##### 4.1.1.3 GliaTrap

GliaTrap has shown strong preclinical promise as a strategy to eliminate the persistent population of glioma cells that escape surgical resection (165, 166). GliaTrap is an injectable hydrogel matrix formulated from polyethylene glycol and hyaluronic acid polymers, cross‐linked via matrix metalloproteinase-sensitive peptide linkers. This design allows the scaffold to conform to the resection cavity geometry and then gradually degrade under physiologic enzymatic activity, minimizing long-term foreign‐body burden (**Figure 4C**). Preclinical evaluation in orthotopic glioblastoma mouse models has demonstrated that a single peri-resection cavity injection of GliaTrap reduces local tumor recurrence by more than 80% compared to surgery alone and extends median survival by approximately 40%. Histopathological analysis confirms that tumor cell accumulation is localized within the dissolving hydrogel, while adjacent neural parenchyma remains unperturbed. Importantly, GliaTrap mesh size and surface functionalization are tuned to preclude non‐neoplastic cell infiltration, thereby minimizing neurotoxicity (166).

Mechanistically, GliaTrap employs a two stage attract-and-kill paradigm. First, the hydrogel releases a controlled gradient of chemokines, principally CCL2 and CXCL12, over several days. These chemotactic signals recruit infiltrative glioma cells into the scaffold interior. Second, once glioma cells have penetrated the network, they encounter immobilized, nanoparticle-bound chemotherapeutics (167). Specifically, PLGA nanoparticles loaded with the chemotherapeutic doxorubicin are tethered within the scaffold via biodegradable linkers. Upon endocytic uptake, the nanoparticles release cytotoxic payloads intracellularly, inducing apoptosis selectively in tumor cells (166).

Safety assessments in both rodent and nonhuman primate studies reveal a favorable profile: neurobehavioral testing indicates no deficits in motor or cognitive function, and systemic pharmacokinetic analyses show minimal doxorubicin leakage into the circulation (166, 168). Collectively, these data position GliaTrap as a highly promising platform for addressing the persistent challenge of infiltrative glioma cells postresection. Its modular architecture also permits the incorporation of additional biologics, such as immunomodulatory cytokines or checkpoint inhibitory antibodies, suggesting broad adaptability for combination therapies in glioma management.

#### 4.1.2 Injectables and CNS-targeting nanotherapeutics for neurodegenerative disease

##### 4.1.2.1 Injectable cytokine releasing hydrogels

Injectable hydrogels formed from natural polymers can encapsulate and gradually release anti-inflammatory cytokines at CNS sites, creating a confined immunoregulatory niche. This strategy minimizes systemic exposure while maintaining therapeutic concentrations in lesioned regions.

Cabré et al. developed a collagen-based hydrogel covalently crosslinked to human IL-10 and stereotactically injected it into the striata of 6-hydroxydopamine-lesioned rats (169). This IL-10 eluting gel remained *in situ* for weeks, markedly reducing microglial activation around dopaminergic grafts and enhancing neuron survival compared to control hydrogels. Their work established proof of concept that hydrogel-mediated cytokine delivery can reshape local inflammation in PD models, inspiring further efforts to tailor gel mechanics and release kinetics.

##### 4.1.2.2 Microglia targeted lipid nanoparticles

Lipid nanoparticles (LNPs) functionalized with targeting ligands (*e.g.*, mannose) can target the delivery of nucleic acids or small molecules to microglia. Badr et al. engineered mannose-coated LNPs carrying an antagomir against microRNA-17 (miR17), which is upregulated in AD microglia (170). Following intracisterna magna injection in 5×FAD mice, these LNPs achieved >90% uptake by microglia, suppressed miR17 expression, restored autophagy pathways, and decreased Aβ plaque burden. Treated animals exhibited reduced proinflammatory cytokines (*e.g.* TNF-α, IL-1β) and significant improvements in spatial memory tasks. This cell-specific approach demonstrates how tailored LNPs can reprogram local immune cells into a neuroprotective phenotype.

##### 4.1.2.3 Implantable cell-isolating scaffolds

Solid scaffolds or cell-laden beads can co-deliver therapeutic cells and immunosuppressants, forming a protected microenvironment that shields grafts from host immunity without systemic immunosuppression.

Atkinson et al. reported an alginate bead system encapsulating human induce pluripotent stem cell (iPSC) derived dopaminergic progenitors alongside tacrolimus-loaded polycaprolactone (PCL) microparticles (171). When delivered into rat striatum, these beads maintained cell viability, reduced T cell activation by 3-fold *in vitro*, and supported neuronal maturation without systemic immunosuppression. The authors optimized bead diameter (~215 μm) for cannula delivery, demonstrating a clinically translatable platform for PD cell therapy that could be adapted to other neurodegenerative contexts.

These biomaterial platforms achieve spatiotemporal control over immunomodulator presentation, fine tuning release profiles, targeting specific immune cell subsets, and co-presenting physical cues that influence phenotypes. By localizing therapy to sites of neurodegeneration, these strategies mitigate peripheral side effects and circumvent BBB limitations. Looking ahead, integration of responsive materials (*e.g.*, ROS or pH-sensitive hydrogels) and multimodal payloads (*e.g.*, combined cytokines and gene therapies) promise even greater precision. Equally, translating these systems into larger animal models and early phase clinical trials will be critical to validate safety, dosing, and long-term efficacy in ALS and AD. As these biomaterial-driven approaches advance, they hold the potential to reshape the treatment landscape for devastating neurodegenerative diseases by harnessing the CNS innate immune capacity for repair under controlled immunological conditions.

#### 4.1.3 Localized neuroinflammation approaches for autoimmunity

##### 4.1.3.1 Injectable DC hydrogels

Injectable hydrogels that deliver tolerogenic dendritic cells provide a localized, sustained platform to modulate neuroinflammation, achieving antigen-specific tolerance in preclinical MS models without systemic immunosuppression. Thomas et al. embedded IL-10–conditioned tolerogenic dendritic cells (DC10s) within a fibrin–alginate hydrogel, stereotactically implanting this depot into the cervical lymph nodes of EAE mice (172). Over four weeks, the gel gradually released DC10s and MCP1, recruiting autoreactive T cells that underwent apoptosis upon encountering DC10s. This localized tolerogenic niche reduced mean paralysis levels and sharply diminished CNS infiltration of Th1/Th17 cells, establishing antigen-specific tolerance without systemic immunosuppression. Beskid et al. similarly demonstrated that IL-10-functionalized PEG hydrogels sustain cytokine release for over two weeks *in vitro*, promoting microglial polarization toward an anti-inflammatory phenotype (173). More recently, Martin Saldaña et al. engineered hyaluronic acid–based hydrogels that modulate neuroinflammation in primary cortical cultures, highlighting matrix stiffness and degradability as tunable parameters to optimize immune cell interactions (174). Liu et al. further reviewed that such injectable gels can be tailored to deliver peptides, nanoparticles, or gene vectors directly into perivascular spaces, circumventing BBB constraints and sustaining therapeutic levels *in situ* (175). Together, these studies underscore how injectable hydrogels can create confined immunoregulatory microenvironments to reeducate autoreactive lymphocytes in MS models.

##### 4.1.3.2 Tolerogenic nanoparticles

Nanoparticle platforms enable precise, antigen-specific immune reprogramming in MS models by codelivering myelin peptides with immunomodulatory cues to peripheral antigen-presenting cells, thereby expanding regulatory pathways and suppressing inflammatory effector responses (176). PLGA nanoparticles provide a modular vehicle for tolerogenic codelivery of antigens and immunosuppressants, generating long-lasting CNS immune tolerance in EAE. Maldonado et al. leveraged 200 nm PLGA nanoparticles co-encapsulating a myelin oligodendrocyte glycoprotein peptide epitope (MOG_35-55_) and rapamycin, administering them intravenously to EAE mice (177). These particles preferentially targeted splenic antigen-presenting cells, expanding MOG specific regulatory T cells over 3-fold while suppressing Th17 differentiation. Treated animals exhibited a relapse rate of 10% versus 80% in free drug controls, with durable protection lasting 60 days post treatment. Nuzzo et al. extended this concept by encapsulating MOG_35–55_ and IL-10 in subcutaneous PLGA nanoparticle depots, reducing peak EAE scores by 70% and delaying onset by one week compared to soluble antigen alone (178). Triantafyllakou et al. explored the combination of mannose and myelin self-antigens to elicit DC uptake, which resulted in enhance therapeutic function in the EAE model (179). Finally, polymerized nanocurcumin nanoparticles attenuated oxidative stress and inflammatory cytokines in EAE, improving locomotor function and myelin preservation (180). These tolerogenic nanoparticle strategies illustrate how codelivery of antigen and immunosuppressant can reprogram peripheral immune cells toward long-lasting CNS tolerance.

MOG-targeted microparticle platforms have also been employed for deletion of pathogenic T cells to restore tolerance, an approach that contrasts with nanoscale systems that rely on intracellular uptake and cellular reprogramming (181). Chen et al. devised 1–3 µm immune-homeostatic microparticles (IHMs) presenting MOG_35–55_ peptides and Fas ligand on their surface (182). A single systemic injection in EAE mice induced apoptosis of pathogenic T cells within lymphoid organs, promoted macrophage‐derived TGFβ secretion, and expanded antigen‐specific Tregs approximately 5-fold. This approach reduced the level of paralysis and preserved myelin integrity, demonstrating a single-dose route to reset immune tolerance. Song et al. have summarized these IHM platforms alongside emerging ROS-responsive delivery systems, emphasizing their capacity for modular payload design and controlled release tailored to inflammatory microenvironments (182).

Although MOG is commonly used in EAE models, it represents only a fraction of CNS myelin and is also implicated in MOG-antibody disease (MOGAD) (183, 184). The platforms in these studies demonstrate that antigen-specific tolerance using biomaterial carriers can be extended beyond MOG to encompass additional clinically relevant autoantigens. For example, Hunter et al. show that PLGA nanoparticles covalently coupled with the myelin proteolipid protein epitope PLP_139–151_ induce potent antigen-specific tolerance in the relapsing-remitting model, preventing and treating relapsing EAE through reduced CNS infiltration of Th1/Th17 cells and suppression of epitope spread (176). Similarly, Saad et al. demonstrate that PLGA nanoparticles encapsulating recombinant human myelin basic protein (MBP) significantly ameliorate EAE severity and neuroinflammation, supporting MBP as a viable tolerogenic cargo beyond MOG-based models. Importantly, the tolerogenic platform is not restricted to T cell–mediated antigens (185). In NMOSD, where pathogenic anti-AQP4 antibodies drive disease, Awad et al. show that AQP4_201–220_-coupled PLGA nanoparticles both prevent and treat disease in a B cell and Th17-dependent model, demonstrating that antigen-specific tolerance using PLGA can be applied to antibody-mediated autoimmunity as well (186). Collectively, these findings broaden the implication of biomaterial-based tolerance strategies by confirming that nanoparticles can effectively deliver diverse CNS autoantigens, including PLP, MBP, and AQP4, to re-establish antigen-specific immune regulation across multiple mechanistically distinct autoimmune diseases.

Collectively, these biomaterial-based therapies deliver payloads to induce durable antigen-specific tolerance while minimizing systemic side effects. Future efforts integrating stimuli-responsive materials and multimodal payloads promise even greater specificity and efficacy in MS treatment.

### 4.2 Systemic mechanism-of-action

Systemic interventions have traditionally faced significant challenges due to the nature of the BBB and the complex immune dynamics of the brain. However, advances in CNS immunity and biomaterial-based therapeutics have enabled targeted, systemic delivery strategies that modulate neuroinflammation. These platforms are designed to circulate systemically and home to vital organs and immune organs to deliver immunomodulatory agents that reprogram immune responses with greater specificity. As a result, systemic biomaterial-based interventions are emerging as promising tools for noninvasive, disease-modifying therapies that harness the immune system to restore homeostasis in neuroinflammatory conditions.

#### 4.2.1 Systemic immunotherapies and vaccines for gliomas

##### 4.2.1.1 Albumin-based nanoparticles for systemic antitumor immunity

Systematically administered albumin-based nanoparticles represent a promising immunotherapy for GBM, and produce therapeutic benefit by selectively crossing the BBB (187). By accumulating in tumor endothelium and immunosuppressive TAMs, they restore antigen presentation and unleash T cell mediated tumor clearance. Gregory et al. developed 100–200 nm albumin‐based synthetic protein nanoparticles (SPNPs) polymerized from human serum albumin (HSA) and decorated with the cell‐penetrating peptide iRGD (188). Intravenous SPNPs loaded with siRNA against STAT3 readily crossed the BBB and penetrated tumors. STAT3 is a key oncogenic and immunosuppressive hub in GBM, and its silencing both slows tumor growth and reverses immune suppression. In mice, STAT3‐silencing SPNPs plus radiotherapy yielded dramatic responses: ~87.5% long‐term survival and formation of anti‐GBM immunological memory. Flow cytometry and histology revealed that this treatment converted the TAM population from an immunosuppressive to an immunogenic state. Immunogenic TAMs rose ~2.5‐fold while immunosuppressive TAMs fell 3- to 4‐fold. Conventional dendritic cell (cDC) antigen presentation was also boosted, and rechallenged survivors showed no tumor recurrence or brain inflammation. These results highlight how nanoparticle‐mediated STAT3 inhibition reshapes the glioma immune milieu. Specifically, the 200 nm iRGD‐SPNPs accumulated preferentially in immunosuppressive TAMs and tumor endothelium, reversed STAT3 signaling, and thereby unleashed T cell–mediated antitumor immunity.

##### 4.2.1.2 Cytokine targeting nanoparticles

Another nanoparticle approach targeted chemokine‐driven immunosuppression. The CXCL12/CXCR4 axis is hyperactive in aggressive GBM and drives MDSC infiltration. Alghamri et al. encapsulated the CXCR4 antagonist AMD3100 into HSA‐based SPNPs bearing iRGD to enhance brain delivery (189). Systemic AMD3100‐SPNPs blocked CXCL12/CXCR4 signaling in GBM models, yielding multiple immunotherapeutic effects. Tumor cell proliferation was halted, and, critically, the influx of CXCR4+ monocytic MDSCs into the tumor was sharply reduced. BBB integrity was improved, and more tumor cells underwent immunogenic cell death (ICD), releasing danger signals that further activate immunity. Combined with radiotherapy, AMD3100‐SPNPs produced durable anti‐GBM immunity, resulting in 60% of mice surviving rechallenge without further therapy. In sum, these ~200 nm iRGD‐SPNPs exploited transcytosis and tumor‐cell homing to deliver small‐molecule inhibitors that dismantle key immunosuppressive circuits in GBM.

##### 4.2.1.3 Nanoparticle-based gene delivery for anti-tumor immunity

Yet another nanoparticle-based strategy is to actively stimulate immunity. For instance, Zhao et al. created biomimetic calcium carbonate nanoparticles cloaked in tumor cell membrane and modified with cRGD peptides (190). These NPs carried mRNA encoding IL‐12, a potent T‐cell–activating cytokine. Systemic injection assisted by ultrasound allowed the NPs to cross the BBB and home to the tumor via cRGD binding and membrane‐homotypic targeting. Upon ultrasound‐triggered release, IL‐12 mRNA was expressed locally, inducing necroptotic tumor cell death and a strong antitumor immune response. Treated tumors showed increased CD8+ T cell infiltration and regression, illustrating how NP‐delivered cytokine gene therapy can convert a low immunogenicity glioma into one susceptible to immunosurveillance.

These examples illustrate that systemic biomaterials for glioma often share common physicochemical features: sizes in the 50–200 nm range specifically balance circulation time and tissue penetration, biodegradable matrices (*e.g.*, albumin, PLGA), and targeting moieties (*e.g.*, RGD peptides, cell‐membrane coatings) breach the BBB and home to tumor sites. By encapsulating immunomodulatory payloads (*e.g.* siRNA, small molecules, mRNA, cytokines) and combining with standard therapy, these NPs leverage both physicochemical crossing of the BBB and biological targeting of immune pathways. Importantly, they are tailored to the unique immunopathology of a glioma by deactivating dominant suppressive pathways and promoting antitumor immunity without broadly ablating systemic immunity.

#### 4.2.2 Systemic immunomodulation to counter neurodegenerative disease

##### 4.2.2.1 Nanoparticle targeting of phagocytic pathways

One strategy for neurodegeneration is to target microglial phagocytic pathways. Gebril et al. devised amphiphilic polymeric NPs that bind microglial scavenger receptors (SRs), enhancing microglial uptake of Aβ fibrils (23). These sugar‐based AM–NPs (50–100 nm) stabilized serum and crossed the BBB; *in vitro* they promoted Aβ clearance and reduced Aβ‐induced microglial activation. *In vivo*, SR‐targeted NPs reduced amyloid burden and neuroinflammation.

Another approach is direct modulation of microglial signaling. Zhang et al. engineered ~200 nm PLGA NPs bearing two functional ligands: the extracellular domain of CD47 (anti-phagocytic signal) attached via a ROS‐sensitive linker, and CRT, a BBB‐penetrating peptide (191). These NPs carried necrostatin‐1 (Nec‐1), a microglia‐modulating drug. In healthy tissue, the CD47 cloak prevents phagocytic clearance, prolonging circulation, while in the diseased brain the CD47 is cleaved, exposing the NPs to microglial phagocytosis. This allows intracerebral delivery of Nec‐1, which inhibits RIPK1‐mediated microglial necroptosis and dampens neurotoxic inflammation. Treated AD‐model mice showed reduced microglial activation and neuronal loss.

##### 4.2.2.2 Inhibiting ROS and oxidative stress

Synthetic nanoparticle nanoenzymes have also been exploited. Ren et al. developed ~5–10 nm quantum‐dot nanozymes with metal oxide cores that spontaneously target microglial mitochondria (192). These nanozymes possess intrinsic antioxidant activity that shift microglia toward an anti-inflammatory phenotype. They readily cross the BBB, escape lysosomes, and accumulate in microglia, and *in vitro* and AD mouse models showed decreased ROS‐driven inflammation and Aβ neurotoxicity. Thus, carefully designed inorganic NPs can serve dual roles as therapeutic agents themselves (*i.e.*, scavenging radicals) and as delivery vehicles.

Polymeric dendrimers offer another paradigm. Hydroxyl‐terminated PAMAM dendrimers (~5–10 nm) inherently accumulate in activated microglia and astrocytes after systemic administration, due to enhanced endocytosis by inflammatory cells. Kannan et al. demonstrated this glia‐homing property by attaching N-acetyl cysteine (NAC) to generation‐4 PAMAM (D‐NAC) (176). In a mouse model of Rett syndrome, D‐NAC intravenous specifically localized in microglia of diseased brains (but not in healthy tissue) and delivered NAC intracellularly. This raised glutathione levels and suppressed oxidative stress and cytokine production, improving neurological outcomes. Similarly, in a rabbit model of inflammatory cerebral palsy, D‐NAC crossed the BBB, replenished microglial glutathione, inhibited NF-κB activation and TNF-α, and markedly reduced neuroinflammation (193). These studies highlight how ~10 nm dendrimer–NAC conjugates exploit size and surface chemistry to achieve cell‐selective CNS delivery.

##### 4.2.2.3 Delivery of anti-inflammatory signals to inhibit neurodegeneration

Other polymeric NPs have been used to deliver anti-inflammatory biologics or genes. For example, Foxp3‐plasmid‐loaded PLGA NPs (100–150 nm) delivered Foxp3 to microglia and significantly reduced inflammatory cytokines (*e.g.*, TNF, IL-1β, IL-6, iNOS) in stroke models. Although not yet applied clinically in AD or PD, such strategies suggest that systemic NPs can reprogram microglia via gene or cytokine delivery. In summary, systemic biomaterials are engineered with features including ultra‐small size, surface ligands or coatings, and biodegradable matrices to traverse the BBB and preferentially engage inflamed neural immune cells. By carrying antioxidants, immunomodulatory drugs, or targeting moieties, these NPs shift microglia away from neurotoxic phenotypes and bolster clearance of pathogenic proteins. Importantly, they work in concert with the known immunopathology of each disease. For example, in AD they may promote Aβ clearance and IL-10/Arg1 expression, while in PD they may inhibit NF-κB and reduce dopaminergic neuron death, and in ALS they could dampen mutant‐protein–induced microglial activation. This disease‐tailored immunomodulation via nanocarriers is an active area of research, with several platforms now entering preclinical testing.

#### 4.2.3 Durable and tolerogenic therapies for autoimmune neuroinflammation

##### 4.2.3.1 Peginterferon β1a (plegridy) in multiple sclerosis treatment

Plegridy is a long-acting, pegylated form of interferon β1a approved for relapsing MS due to its sustained clinical benefit and substantially reduced injection burden. Pegylation enhances drug half-life and systemic exposure, enabling subcutaneous or intramuscular dosing once every two weeks, a marked improvement over the frequent dosing required with earlier interferon-based therapies (194, 195). In the pivotal ADVANCE trial, Plegridy produced meaningful reductions in relapse rates and MRI lesion activity, and these therapeutic gains were maintained over several years in extension studies (196). This prolonged systemic delivery provides continuous immunomodulation, targeting the neuroinflammation that drives MS disease activity (**Figure 5A**).

As a type I interferon, Plegridy binds to interferon receptors on immune cells, activating JAK–STAT signaling and inducing interferon-stimulated genes. These effects shift the immune response away from pro-inflammatory Th1 activity by reducing cytokines such as TNF-α and IFN-γ and downregulating MHC class II on antigen-presenting cells (197). Plegridy also upregulates anti-inflammatory cytokines (*e.g.* IL-4, IL-10), decreases adhesion molecule expression, stabilizes the BBB, and limits leukocyte infiltration into the CNS. In B cells, it promotes apoptosis, thereby reducing pathogenic memory B cell populations implicated in MS (198).

Clinical trials have demonstrated Plegridy efficacy and durability. In the Phase III ADVANCE trial, biweekly 125 μg dosing reduced the annualized relapse rate by approximately 36% and new T2 lesion count by 67% versus placebo at one year (196). Plegridy also lowered the risk of confirmed disability progression by 38%. Long-term extension studies, such as ATTAIN, confirmed sustained reductions in relapses and lesion accumulation for up to six years, with many patients achieving no evidence of disease activity (199). Compared with other interferon-based therapies, the prolonged action, lower injection frequency, and low immunogenicity (<1% neutralizing antibody formation) contribute to improved adherence and outcomes for Plegridy (195, 200).

Plegridy is generally well tolerated, with adverse effects similar to conventional interferons, including immunosuppression, flu-like symptoms, injection-site reactions, and mild laboratory abnormalities. While newer MS therapies may offer higher efficacy in aggressive disease, the favorable safety profile and convenience of Plegridy make it a key systemic strategy for long-term modulation of neuroinflammation in MS.

##### 4.2.3.2 Lymph node engineering for immune tolerance

Pioneering work on lymph node engineering through creation of biomaterial-based drug depose has enabled regulation of antigen-specific immune tolerance in models of MS and other autoimmune diseases (201, 202). Unlike systemic immunosuppression, this technique uses direct lymph node delivery of PLGA microparticles encapsulating MOG_35-55_ peptide and rapamycin, promoting antigen-specific tolerance with minimal systemic exposure (**Figure 5B**) (201). Injected under ultrasound guidance or with the use of tracer dyes, these microparticles remain localized, enabling sustained release and synergistic signaling to expand regulatory T cells (Tregs) and reduce inflammatory cytokines such as IL-17 and IFN-γ (201).

This localized approach induces systemic tolerance through Tregs migrating into peripheral tissues and the CNS, markedly reducing infiltration by autoreactive T cells and protecting myelin integrity. In mouse EAE models, a single intralymphatic injection at peak disease fully reversed paralysis within 48 hours and prevented relapse long-term (203, 204). Safety assessments indicated minimal local toxicity, low systemic rapamycin levels, and preserved immune responses against unrelated antigens and pathogens, clearly distinguishing this from traditional immunosuppressive treatments (205).

Compared to conventional systemic therapies that broadly suppress immunity, the lymph node engineering approach offers antigen-specificity, lower systemic drug exposure, and the potential for single-dose durability. Ongoing research focuses on improved biomaterial formulations and minimally invasive delivery methods, supporting the viability of intralymphatic biomaterial depots as precise, durable therapies for MS.

##### 4.2.3.3 Immunosuppressive vaccines

Recent advances demonstrate that systemic administration of biomaterial-based vaccines can re-establish antigen-specific immune tolerance in chronic MS. Nguyen et al. engineered mesoporous silica nanoparticles (MSNs) loaded with MOG_35-55_ peptide and decorated with ROS–scavenging cerium oxide nanoparticles (CeNPs), enabling intravenous delivery of a self-antigen/CeNP nanovaccine that homes to splenic APCs (**Figure 5C**) (181). Upon systemic injection, MOG-MSNs accumulate in CD11c+ dendritic cells, F4/80^+^ macrophages, and B220^+^ B cells, where slow release of peptide undercuts costimulatory molecule upregulation and favors semimature, tolerogenic APC phenotypes. Decoration with CeNPs further suppresses intracellular ROS and CD86+CD40+ expression on APCs even in pro-inflammatory environments, amplifying tolerogenic signaling.

This coordinated antigen delivery and redox modulation drives peripheral expansion of Foxp3+ Tregs while reducing pathogenic IL-17A, GM-CSF, and TNF-α secretion upon antigen recall. Critically, systemic tolerance established in spleen translates into profound protection of the CNS. MOG-MSN–Ce vaccination reduces CNS infiltration by autoreactive CD4+ T cells and APCs, suppresses microglial activation, and reverses paralysis even when administered in late chronic EAE models. Single-dose nanovaccine therapy yields durable remissions without broad immunosuppression, minimal systemic Ce or MOG leakage, and preserved responses to unrelated antigens, distinguishing it from conventional therapies (181).

By converting systemic delivery into a targeted, antigen-specific tolerogenic intervention, this MSN/CeNP platform exemplifies how biomaterials can reshape peripheral immunity to halt neuroinflammation and demyelination in MS. Future work will refine nanoparticle composition, dosing regimens, and translational scaling to human lymphoid targeting, underscoring the promise of systemic biomaterial-based vaccines as next-generation MS therapeutics.

### 4.3 Comparative safety profiles of biomaterial and systemic therapeutic approaches

Biomaterial delivery platforms can markedly reduce the off-target toxicities of systemic immunotherapies by concentrating antigen or immunomodulatory cargo in tolerogenic tissues or the diseased microenvironment, enabling lower total doses, controlled release, and biomimetic presentation that better recapitulates apoptotic-cell-driven tolerance (206, 207). These advantages have supported translation of polymeric carriers into clinical development and have been linked to improved therapeutic windows for otherwise toxic payloads. Importantly, however, particle design governs risk: surface-displayed antigens or certain chemistries can raise immunogenicity and, in rare cases, provoke severe hypersensitivity (including anaphylaxis), whereas encapsulation strategies tend to blunt immediate antigen recognition and are therefore often safer *in vivo* (206, 208). In conclusion, the literature supports that biomaterials can improve precision and reduce systemic immunosuppression, but clinical success requires deliberate control of antigen density, route, particle composition (e.g., encapsulated *vs* surface-coupled), and dosing to mitigate immunotoxicity while preserving tolerogenic function.

## 5 Safety and clinical translation

Although CNS-targeted biomaterials continue to show strong potential, they still face major limitations on both the regulatory and biological aspects. One of the biggest challenges is that regulatory approvals are tied very closely to specific indications and delivery routes. For example, the Gliadel wafer is approved for high-grade glioma, and that approval does not automatically translate to other CNS conditions (155, 156). At the same time, previous approvals can still be helpful because they provide precedent and can make it easier to justify the safety of a biomaterial in a new context. Biology adds another level of complexity. Different CNS diseases involve very different microenvironments, including unique patterns of inflammation, scarring, tissue stiffness, and BBB disruption, so a material that performs well in one setting may behave unpredictably in another (209). Preclinical systems, from *in vitro* BBB models to animal studies, often fail to fully mimic the human brain, which contributes to frequent setbacks during translation.

Looking forward, several developments may help address these barriers. Regulatory agencies are moving toward more unified approaches to evaluating nanomedicine and biomaterials, and early engagement with regulators can help teams identify clearer approval pathways (210). Importantly, successful products can serve as precedents that demonstrate the feasibility of intracranial biomaterial delivery, but progress will also depend on improving standardization.

New delivery strategies are another promising direction. Approaches such as intranasal nose-to-brain delivery, receptor-mediated BBB transport, focused ultrasound-based BBB opening, and implantable hydrogel systems are being actively explored to improve access to brain tissue. These methods often pair well with imaging and neurosurgical techniques, offering more precise control over biomaterial trafficking and biodistribution. Artificial intelligence (AI) approaches are also beginning to play a role (211). Machine learning tools can analyze large datasets to predict which polymers, nanoparticle designs, or scaffold features are most compatible with neural tissue (211). Although still in early stages, these computational methods could help reduce trial-and-error and speed up biomaterial discovery. Overall, translating biomaterial therapies for CNS disorders will require navigating both the biological diversity of different diseases and the complexity of the regulatory landscape. While each indication demands rigorous evidence of safety and efficacy, existing clinical platforms show that intracranial biomaterials can be used safely when properly designed. Continued progress in standardization, manufacturing, and advanced delivery methods, combined with interdisciplinary collaboration, will help bring more of these technologies into the clinic (212, 213). As new tools, including AI-guided design, begin to shape biomaterial development, the field is well positioned to expand the impact of biomaterial therapies across a wide range of neurological diseases.

## 6 Conclusion

This review highlights neuroinflammation as a common thread in CNS diseases, and that engineered interventions can capitalize on neuroimmune regulation for diagnosis and therapy. In gliomas, chronic inflammation paradoxically promotes tumor growth and immune evasion. Glioma cells secrete factors that recruit macrophages/microglia and polarize them into an immunosuppressive state. Consequently, TAMs and neutrophils produce angiogenic and trophic factors, while effector T cells are rendered exhausted by checkpoint ligands (*e.g.*, PD-L1) and suppressive cytokines. The net result is unchecked tumor expansion and treatment resistance.

Neurodegenerative diseases (*e.g.*, AD, PD, ALS) provide a contrasting outcome of chronic inflammation. Here, age- or mutation-driven protein aggregates incite microglial activation that initially attempts cleanup but becomes self-perpetuating. Persistently activated microglia and astrocytes release neurotoxic cytokines (*e.g.*, IL-1β, TNF-α) and reactive oxygen/nitrogen species. Over time this inflammation worsens protein pathology and synaptic loss, as in AD where microglial IL-1 accelerates tau phosphorylation. Adaptive immune cells also play roles in neurodegenerative disease. For example, autoreactive T cells recognizing α-synuclein may infiltrate PD brains and kill neurons. As a result, neurodegenerative outcomes are driven by glial dysfunction and secondary immune recruitment, fueling a feedback loop of neuronal injury.

Finally, MS exemplifies neuroinflammation driven by peripheral autoimmunity. In MS, activated autoreactive T cells, myelin-targeting antibodies, and inflammatory innate cells breach the BBB to coordinate attacks on myelin. Early BBB breakdown allows infiltrating lymphocytes (*e.g.*, Th1/Th17 cells) to enter the CNS. Inflammatory macrophages and microglia then perpetuate damage by phagocytosing myelin and releasing cytotoxic mediators. Over repeated relapses, oligodendrocytes are lost and axons degenerate, leading to irreversible disability. The outcome is demyelination and neurodegeneration fueled by both innate and adaptive immune dysregulation. Collectively, these sections underscore that neuroimmune regulation is inherently multifaceted with distinct opportunities for diagnosis and intervention.
